# Structure-Based Mechanism and Specificity of Human Galactosyltransferase β3GalT5

**DOI:** 10.1021/jacs.4c11724

**Published:** 2025-03-25

**Authors:** Jennifer M. Lo, Chih-Chuan Kung, Ting-Jen Rachel Cheng, Chi-Huey Wong, Che Ma

**Affiliations:** † Genomics Research Center, Academia Sinica, Taipei 115, Taiwan; ‡ Chemical Biology and Molecular Biophysics Program, Taiwan International Graduate Program, Academia Sinica, Taipei 115, Taiwan; § Department of Chemistry, National Tsing Hua University, Hsinchu 300, Taiwan; ∥ Department of Chemistry, Scripps Research, La Jolla, California 92037, United States

## Abstract

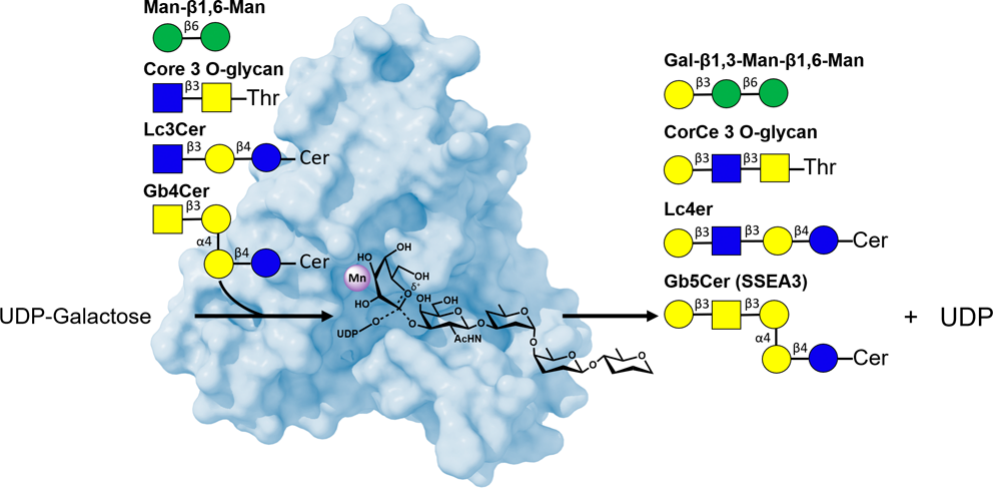

Human β1,3-galactosyltransferase 5 (β3GalT5) is a key enzyme involved in the synthesis of glycans on glycoproteins and glycolipids that are associated with various important biological functions, especially tumor malignancy and cancer progression, and has been considered as a promising target for development of anticancer agents. In this study, we determined the X-ray structures of β3GalT5 in complex with the stable donor analogue UDP-2-fluorogalactose or the native donor substrate UDP-galactose (UDP-Gal) and several glycan acceptors at different reaction steps. Based on the structures obtained from our experiments, β3GalT5 catalyzes the transfer of galactose from UDP-Gal to a broad spectrum of glycan acceptors with an S_N_2-like mechanism; however, in the absence of a glycan acceptor, UDP-Gal is slowly converted to UDP and two other products, one is galactose through an S_N_2-like mechanism with water as an acceptor and the other is an oxocarbenium-like product, presumably through an S_N_1-like mechanisms. The structure, mechanism, and specificity of β3GalT5 presented in this study advance our understanding of enzymatic glycosylation and provide valuable insights for application to glycan synthesis and drug design targeting β3GalT5-associated cancer.

## Introduction

Human beta-1,3-galactosyltransferase 5 (β3GalT5) is a member of the glycosyltransferase-31 family which is expressed in various tissues^1,2^ and catalyzes the β-1,3-galactosylation of glycans on glycoproteins and glycolipids. β3GalT5 accepts glycans with terminal galactose (Gal), *N*-acetylglucosamine (GlcNAc), or *N*-acetylgalactosamine (GalNAc), leading to various important biological functions, including development, immune response, and cancer progression.^3−7^ While other family members of β3GalT show different substrate specificity for N- and O-linked glycans, β3GalT5 prefers core 3 structures, globo- and Thr O-glycans, as well as type 1 Lewis glycans as substrates,^2,7,8^ and the products generated are often associated with tumor malignancy and cancer progression, thus termed as tumor-associated carbohydrate antigens (TACAs).

**Figure 1:**
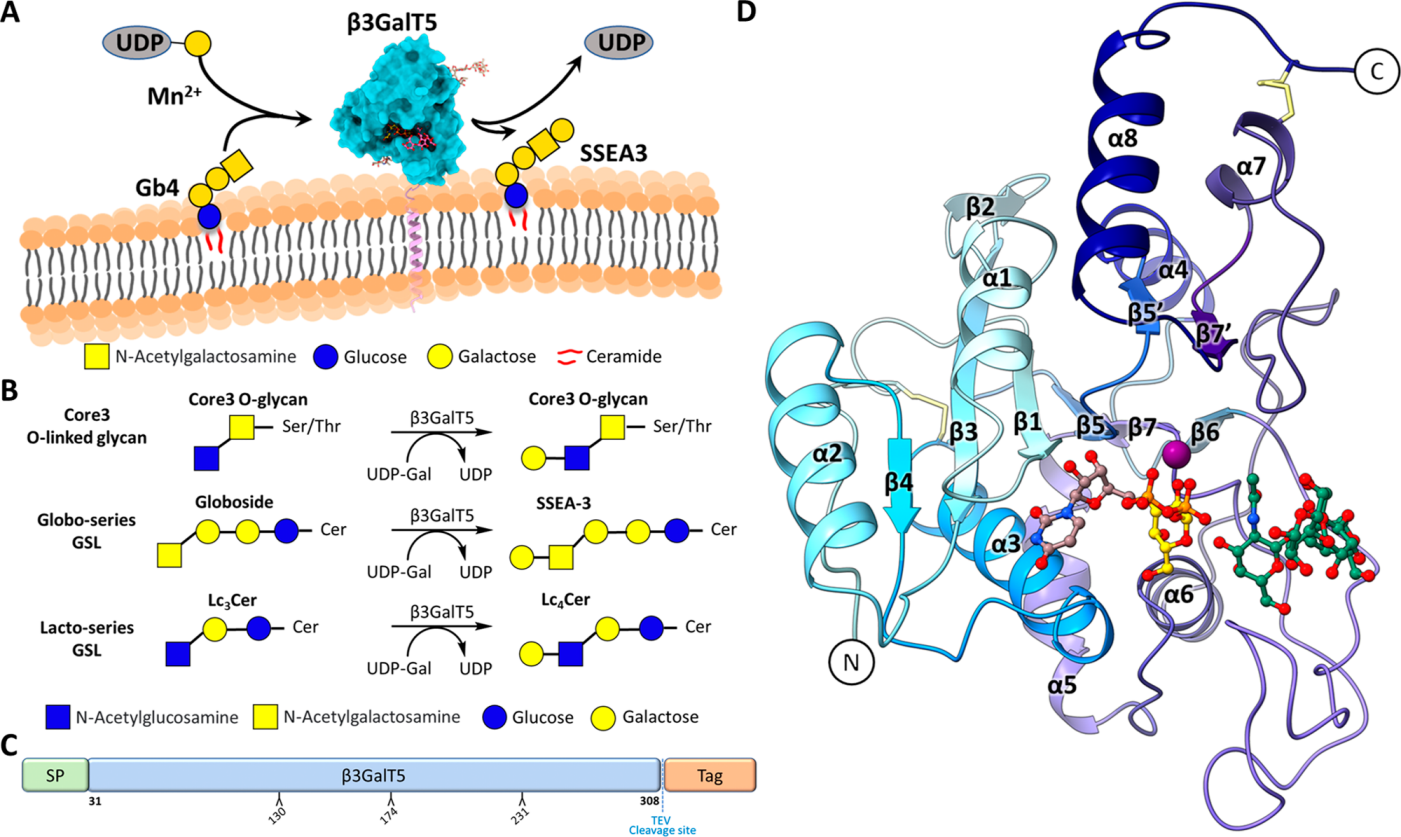
Overall structure of human β3GalT5. (A) Synthesis of SSEA-3 from Gb4 catalyzed by β3GalT5. (B) β3GalT5 catalyzed galactosylation in the synthesis of Core 3 O-glycans, globo-series glycosphingolipids (GSLs), and lacto-series GSLs. (C) Construct of gene for β3GalT5 expression. (D) Overall structure of β3GalT5 with substrates: composite image with β3GalT5:UDP2FGal superimposed on β3GalT5:Gb4 glycan. The color gradient from light to dark blue indicates the protein structure from N-terminal to C-terminal. The divalent ion Mn^2+^ is colored purple, UDP is brown, donor sugar galactose is gold, and acceptor Gb4 glycan is green.

It has been observed that high expression of β3GalT5 correlates with advanced cancer progression and poor clinical outcome in breast,^3,5,9^ pancreatic,^10,11^ liver,^4,7^ ovarian,^12^ gastric,^13^ and nonsmall cell lung^14^ cancers and that β3GalT5 promotes cancer cell proliferation, migration, and invasion by regulating the expression of cell adhesion molecules and extracellular matrix proteins. For example, β3GalT5 catalyzes the galactosylation of type 1 chain *N*-acetyllactosamine glycan (GlcNAc-β1,3-Gal-) (Figures 1B and S1) to form Lewis a (Lea), Lewis b (Leb), and sialyl Lewis a (sLea). Notably, sLea, also known as carbohydrate antigen 19-9 (CA19-9), serves as a useful tumor marker for the detection of early stage cancer and is frequently accumulated in the sera of patients with colonic, gastric, and pancreatic cancer.^10,15^ CA19-9 was also found to accelerate cancer progression through the PI3K/Akt/mTOR pathway and selectin-mediated signaling.^10^ In the biosynthesis of globo-series glycosphingolipids, β3GalT5 catalyzes the transfer of Gal from UDP-Gal to the terminal GalNAc residue of globotetraosyl ceramide (Gb4cer) (Figures 1A and S1) to generate Gb5 (also known as stage-specific embryonic antigen-3, SSEA-3). SSEA-3 can be further converted to SSEA-4 or Globo-H. Many cancers exhibit enhanced expression of SSEA-3, SSEA-4, and Globo-H, making these three cancer-associated globo-series glycosphingolipids (GSLs) promising clinical targets for immunotherapy.^9,16−19^ A recent study showed that SSEA-3, SSEA-4, and Globo-H formed a complex with FAK/CAV1/Akt/RIP for signaling through the AKT-associated EGFR pathway and promoted cancer cell survival and metastasis.^16^ Knockdown of β3GalT5 expression in breast cancer cells caused dissociation of RIP from the complex, triggering cancer cell apoptosis.^16^ Additionally, silencing β3GalT5 reduces the migration and invasion ability through the regulated β-catenin/ZEB1 pathway during the epithelial-to-mesenchymal transition process. In vivo studies using patient-derived xenograft transplanted mice models demonstrate that β3GalT5 not only promotes tumor growth but also stimulates lymph node and lung metastasis.^5^

We also demonstrated that the globo-series glycans are exclusively expressed in 15 types of cancers and their stem cells^20^ and knockdown of β3GalT5 led to cancer cell apoptosis with no effect on normal cells.^16^ This work has led to the development of therapeutic cancer vaccines targeting Globo-H and the other globo-series glycans.^21^ Taken together, β3GalT5 plays a pivotal role in the synthesis of TACAs and is considered as a promising target for development of anticancer agents. In this study, we determined the X-ray structure of the luminal domain of human β3GalT5 and elucidated its mechanism and specificity with the goal of providing valuable guidelines to facilitate the development of structure-based anticancer agents. We determined the structures of human β3GalT5 at various stages (a total of 8 structures) of the enzymatic reaction, including the dissociated galactose transition-like structures, and identified a broad-spectrum acceptor substrate, manifested by detailed structural rationales of each step for elucidation of the reaction mechanism and laying the groundwork for rational drug design and better understanding of glycobiology.

## Results and Discussion

### Overall Structure of β3GalT5

β3GalT5 is a member of the glycosyltransferase-31 (GT31) family of carbohydrate-active enzymes (CAZy)^22^ and exhibits limited overall sequence identity (25–29%) to other family members. This limited sequence homology has hindered the establishment of robust structure–function relationships.^8^ To address this issue, we expressed and purified β3GalT5 and cocrystallized it in the presence of UDP-galactose (UDP-Gal) and different substrates. Our structural investigations began by solving the luminal domain structure of human β3GalT5 using sulfur single-wavelength anomalous dispersion phasing, achieving a resolution of 2.20 Å (Figure S2A–E). Subsequently, we determined additional β3GalT5 structures in complex with various substrates or products using molecular replacement with the sulfur derivative structure serving as the template. In total, we obtained eight structures, including β3GalT5 in complex with UDP-Gal, Gb4 glycan, GlcNac-β1,3-Gal, GlcNAc-β1,3-GalNAc, and Man-β1,6-Man. All structures belong to the P1 21 1 space group, with each asymmetric unit containing two protein molecules (Figure S2F).

β3GalT5 contains 310 amino acids, including the cytosolic N-terminal sequence (residues 1–7), the transmembrane domain (residues 8–28), and the soluble catalytic domain (residues 29–310). The recombinant protein used for crystallization contains residues Phe31-Pro308 (Figure 1C), but only residues Asp41-Pro308 are well-defined in the electron density maps. The catalytic domain adopts a mixed α/β Rossmann-like fold commonly observed in the GT-A glycosyltransferases superfamily and consists of a seven-stranded β sheet core (β1, β2, β3, β4, β5, β6, and β7) surrounded primarily by α helices (α1, α2, α3, α4, α5, α6, and α7), a two-strand antiparallel β sheet (β5′ and β7’),^23^ and an additional 13-residue α helix (α8) at the C terminus (Figure 1D). Notably, two disulfide bonds (Cys52-Cys146 and Cys276-Cys307) stabilize the structure, and of the three glycosylation sites (Asn130, Asn174, and Asn231), the Asn174 glycosite reveals a complete paucimannose structure with one core fucose, which stabilizes the crystal packing by anchoring between adjacent molecules (Figure S2G).

Regarding the structural homology of β3GalT5 to other described structures, the DALI server^24^ reveals structural similarities with other galactosyltransferases, including β1,3-*N*-acetylglucosaminyltransferase 2 (B3GNT2; e.g., PDB entries 6WMO,^25^7JHN,^26^8TJC^27^), *D. melanogaster* core 1 synthase glycoprotein-*N*-acetylgalactosamine 3-β-galactosyltransferse 1 (*Dm*C1GalT1; PDB entries 7Q4I(28)), mouse manic fringe (Mfng; PDB entries 2J0A and 2J0B(29)), and *A. fumigatus* galactofuranosylransferase (*Af*GfsA; PDB entries 8YRL), all of which belong to the CAZy31 family. Despite their diverging sequence identity to β3GalT5 (29, 19, 17, and 13%, respectively) and differing acceptor substrates, the server yields favorable scores, suggesting good structural superimposition (rmsd of ∼1.7, ∼2.1, ∼2.3, and ∼3.0 Å, respectively; with superimposed residues ranging from 112 to 203 residues). For retaining GT, α-1,3-galactosyltransferase (α3GalT; PDB entries 5NRD(30)) also has comparable key interacting residues to those of inverting GTs. The rmsd value is around 3.9 Å when compared to β3GalT5 with 90 Cα-atoms. The common active site architecture can be observed among these structures, despite differences in their substrate binding and reaction mechanisms (Figure S3).

### Substrates Binding Clefts

**Figure 2:**
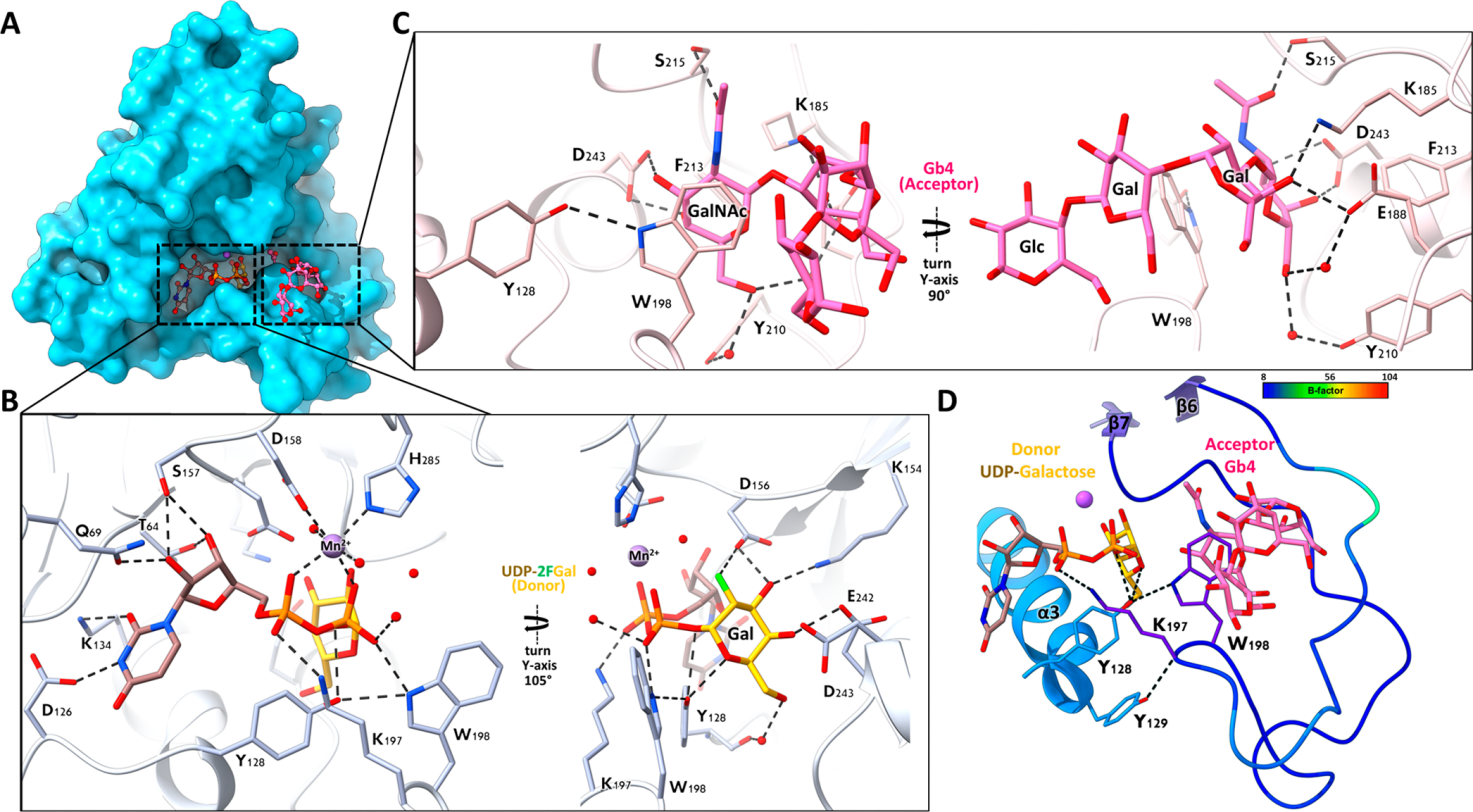
Donor and acceptor binding clefts in β3GalT5. (A) Surface representation of the β3GalT5 structure, showing the substrate-binding cleft with donor on the left-hand side and acceptor on the right-hand side. (B) Binding cleft for UDP-2FGal and the interacting residues. The left figure shows UDP interactions, and the right figure shows 2F-galactose interactions. Hydrogen bonds are represented by dotted lines. UDP is colored brown and galactose colored gold. (C) Binding cleft for acceptor Gb4 glycan and interacting residues is shown. Hydrogen bonds are represented by dotted lines. Gb4 glycan is colored hot pink. (D) Residues K197 and W198 on the long loop between β6 and β7 sheets interact with residues Y128 and Y129 on α3 helix that form the acceptor binding cleft. The long loop is colored based on residue’s Cα *B*-factor (average values of 16.76 Å^2^) with blue to red. The Cα *B*-factors are depicted on the whole protein structure in blue (lowest *B*-factor, 8.03 Å^2^) to red (highest *B*-factor, 104.36 Å^2^).

Both donor and acceptor sugars are located close to the center of the whole binding cleft (Figure 2A). During the cocrystallization of UDP-Gal and β3GalT5, the electron density did not show the presence of galactose (Gal) covalently linked to UDP. This absence is likely due to the hydrolysis of the UDP-Gal donor during crystal formation. To circumvent this issue and obtain structural information on the UDP-Gal binding cleft, we employed an inert analog of the sugar nucleotide donor, UDP-2-deoxy-2-fluoro-α-D-galactopyranose (UDP-2FGal) for cocrystallization with β3GalT5. The replacement of Gal with 2FGal stabilized the donor sugar from hydrolysis.^28^ In the enzyme-UDP-2FGal complex, the UDP moiety binds with the divalent ion Mn^2+^ and the side chain of Tyr128, Lys154, Asp156 (Asp from the DxD motif), and Glu242 (xED motif commonly observed in inverting galactosyltransferase) is hydrogen-bonded with the 2FGal moiety with Tyr128 engaged in additional interaction with the O1 (Figure 2B). These interactions force the 2FGal moiety to place directly above the pyrophosphate moiety in the tucked conformation required for catalytic activity.^31^ The side chain of 2FGal was held in the *trans*–*gauche* (tg) conformation consistent with the pattern for a β-galactosyltransferases.^32^ The donor binding cleft contains Asp158 in the Asp-Ser-Asp (DxD motif) triplet and His285, which is used to coordinate with the diphosphate of UDP together with two water molecules through a Mn^2+^ divalent cation in an octahedral geometry. The DxD motif is commonly observed in metal-dependent GT-A fold enzyme^33^ (e.g., DSD for β3GalT5, DDD for β3GNT2,^25,26^ Mfng,^29^ and C1GalT1,^28^ and DVD for α3GalT^30^). For the UDP moiety, the side chain of Asp126 and Lys134 interacted with the uracil N3 and O2 atoms, respectively; whereas the side chain of Thr64, Gln69, and Ser157 was hydrogen-bonded to the ribose hydroxyls. The side chain of Tyr128 and Lys197 interacted with the pyrophosphate group of UDP (Figure 2B). Specifically, Lys197 was at the long loop (Lys172-Thr217) between β6 and β7, its side chain formed a salt bridge with the α-phosphate oxygen atom, and the main chain interacted with the side chain of Tyr129 at α3. These interactions provided a significant contribution to keep the loop closed, thus facilitating further interaction between Trp198 NE1 and the hydroxyl group on the side chain of Tyr128 to form the acceptor binding cleft for UDP-Gal-Mn^2+^ (Figure 2D). This strategically arranged loop can prevent excess water from reaching the enzyme’s active site and creates an environment to lower the transition state energy in catalysis and to form a lid over the nucleotide binding site allowing acceptor binding and facilitating the reaction.^34,35^

For the acceptor binding cleft, we observed that β3GalT5 only interacted with the nonreducing end N-acetylgalactosamine (GalNAc) and the following galactose from the structure of β3GalT5 in complex with UDP-Gal and Gb4 glycan. The acceptor GalNAc is stacked against the side chains of Trp198 and Phe213, which placed the acceptor sugar into the catalytic binding cleft. The residue Asp243 (xED motif) further interacted with 3-OH and 4-OH of the acceptor GalNAc. This position structurally aligns well with other GT-A fold enzymes (e.g., D333 in β3GNT2,^25,26^ D232 in Mfng,^29^ D255 in C1GalT1,^28^ D335 in GfsA, and α3GalT, which is switched to xDE^30^). Ser215 formed a hydrogen bond with the acetamide carbonyl, and Glu188 and Tyr210 formed a water-mediated hydrogen bond interaction with the 6-OH of GalNAc. Lys185 and Glu188 were the only two residues that interacted with the following galactose (Figure 2C). The acceptor binding moiety has relatively fewer interactions in comparison to the donor binding moiety, resulting in a wider tolerance to different acceptors (Figure S1). This is in line with our kinetic study, in which the binding of UDP-Gal was around 12-fold stronger than the binding of Gb4 glycan.

### Substrate Specificity

While β3GalT5 selectively binds to UDP-Gal as the donor substrate (Figure S4), it exhibits broader selectivity for acceptor substrates (Figure S1). Specifically, β3GalT5 catalyzes the transfer of galactose from UDP-Gal to both the Gb4 glycan (with GalNAc at the nonreducing end) and Lc3 glycan (with GlcNAc at the nonreducing end) via a β1,3-linkage. Using disaccharides as substrates and measuring the end point UDP concentration after 1 h reaction time, we have selected Man-β1,6-Man and GlcNAc-β1,3-GalNAc-α-Thr in addition to Gb4 glycan and GlcNAc-β1,3-Gal-OMe for further structural studies of the enzyme–substrate complex obtained from β3GalT5 cocrystallized with UDP-Gal and soaked with various acceptors. The density maps for the substrates are shown in Figure S6.

**Figure 3:**
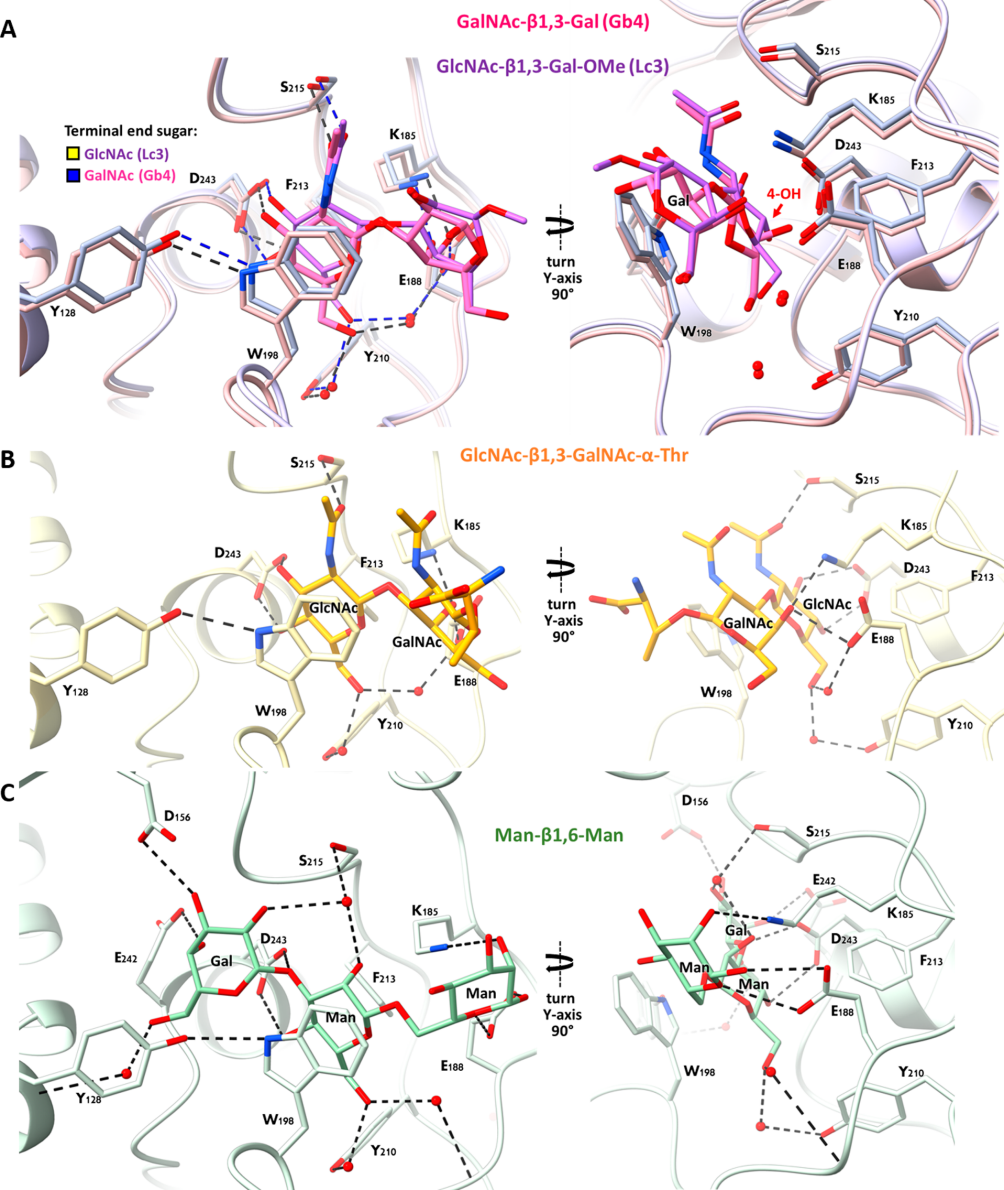
Wide-spectrum acceptor substrate tolerance of β3GalT5. The structures are obtained by cocrystallizing β3GalT5 with UDP-Gal, followed by soaking with different acceptors. (A) Superimposition of Gb4 glycan (in pink; with only the terminal disaccharides for comparison) and the disaccharide GlcNAc-β1,3-Gal of Lc3 (in purple) ligand-bound structures reveals similar interactions. (B) GlcNAc-β1,3-GalNAc-α-Thr (in orange) acceptor binding cleft exhibits identical interactions when compared to the Gb4 glycan bounded structure. (C) Man-β1,6-Man (in green) acceptor ligand-bound structure indicates the formation of the product Gal-β1,3-Man-β1,6-Man. The interacting residues are identical to those observed in other acceptor bound structures with flexible substituents at C2 and C4 and additional interactions with water and product. Hydrogen bonds are represented by dotted lines, and sugars are labeled. The omit maps are shown in Figure S6.

The distinction between GalNAc and GlcNAc lies in the position of the 4-OH group. In the ternary structure of β3GalT5:UDP:GlcNAc-β1,3-Gal-OMe (a disaccharide derived from Lc3), the nonreducing end GlcNAc exhibits interactions similar to those observed with GalNAc. Despite the difference in the 4-OH position, the amino acid Asp243 maintains hydrogen bonding with both the 3-OH (2.57 Å for GlcNAc and 2.89 Å for GalNAc) and 4-OH (2.72 Å for GlcNAc and 2.76 Å for GalNAc) groups. The distances between GlcNAc and Asp243 are shorter, potentially resulting in a lower *K*_m_ value (0.5 mM for GlcNAc-β1,3-Gal-OMe and 2.0 mM for Gb4 glycan). Furthermore, the adjacent galactose residue also engages in comparable interactions. Hence, there are no significant differences in structural conformation (rmsd of 0.10 Å for 234 Cα-atoms) (Figures 3A and S5A,B).

The discovery of Man-β1,6-Man and GlcNAc-β1,3-GalNAc-α-Thr as substrates with comparable end point UDP concentration after 1 h reaction with Gb4 and Lc3 glycans is intriguing. Notably, the interaction pattern between β3GalT5 and GlcNAc-β1,3-GalNAc-α-Thr mirrors that observed in interactions with Lc3 glycan (Figure 3B). There are no direct interactions between β3GalT5 and the 2-OH group of galactose in the disaccharides of Gb4 or Lc3. Since the galactose moiety protrudes into the open space, its replacement with GalNAc-α-Thr is unlikely to significantly impact the overall structure (with an rmsd of 0.14 Å for 229 Cα-atoms) (Figure S5A). The *K*_m_ value for GlcNAc-β1,3-GalNAc-Thr is 0.75 mM, similar to that for GlcNAc-β1,3-Gal-OMe (Figure S5B).

Surprisingly, we observed comparable enzyme end point activity for the mannose disaccharide with an β1,6-linkage, and we detected a product with galactose transferred onto the substrate. Notably, there is no significant difference in protein levels when comparing the Gb4 glycan and Man-β1,6-Man-bound structures (with an rmsd of 0.15 Å for 238 Cα-atoms). The dissimilarities at the terminal end of mannose involve the glycosidic bond between 3-OH and galactose, water-mediated interactions between Ser215 and 2-OH of mannose and galactose, and the absence of a water-mediated interaction between 6-OH and Glu188. For the reducing end mannose, the 2-OH interacts with Lys185 and Glu188 interacts with 1-OH and O5 (Figure 3C). Consequently, Man-β1,6-Man exhibits more water-mediated interactions and fewer amino acid direct interactions. Additionally, the β1,6-glycosidic bond between mannose may also contribute to the recognition of such a disaccharide substrate. Further kinetic analysis revealed that the *K*_m_ value for the Man-β1,6-Man is 2.5 mM, which is the highest among the four substrates and correlates with the structural observation (Figure S5B).

### Mechanism of β3GalT5 Inverting Galactosyltransferase

The glycosylation reactions, which involve the nucleophilic substitution of a leaving group in a glycosyl donor with a glycosyl acceptor, can occur via an S_N_2 or S_N_2-like mechanism, a stepwise S_N_1 or S_N_1-like mechanism involving oxocarbenium intermediates, or S_N_*i* mechanisms involving tightly or loosely associated ion pairs, where nucleophilic attack occurs at the same face as the leaving group. Most inverting glycosyltransferases (GTs) are suggested to utilize a displacement mechanism with an oxocarbenium ion-like transition state that happens concurrently in an S_N_2-like reaction. In contrast, glycosyl oxocarbenium-like intermediates in a double displacement mechanism are plausible for those enzymes that operate through S_N_1 and S_N_*i* mechanisms. It often involves the formation and subsequent breakdown of a covalent glycosyl–enzyme intermediate in which a nucleophile is required for attack on the anomeric center of the donor sugar to form the glycosyl–enzyme species.^36−42^

**Figure 4:**
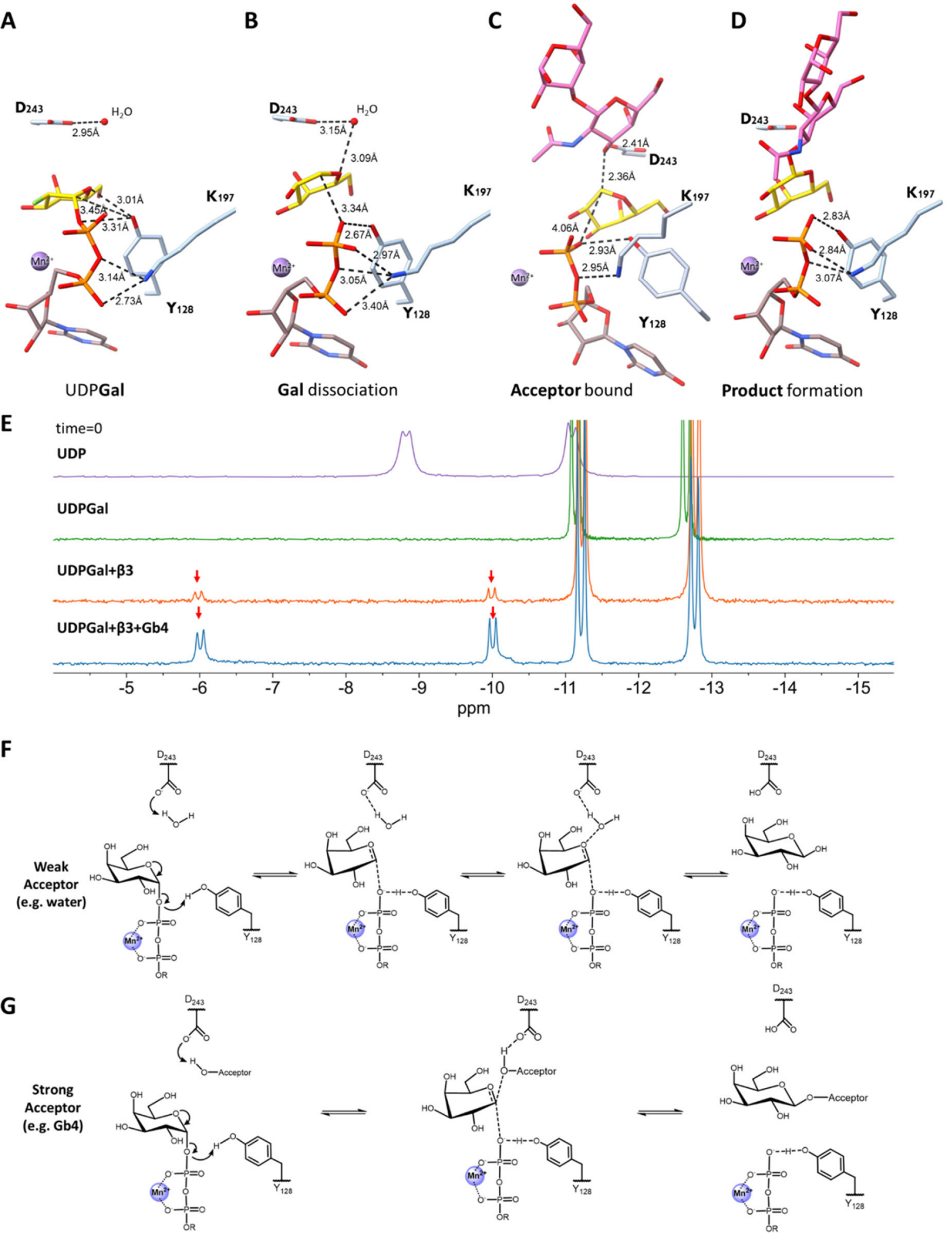
Proposed glycosylation mechanism catalyzed by β3GalT5. (A) β3GalT5 cocrystallized with the UDP-2FGal structure represents the interacting groups in the purposed mechanism (PDB: 8ZWR). (B) β3GalT5 cocrystallized with the UDP-Gal structure represents the purposed mechanism where the glycosidic bond between UDP and galactose is being cleaved (PDB: 8ZX9). (C) β3GalT5:UDP-Gal:Gb4 glycan ternary structure represents the purposed mechanism where Gb4 glycan bound to the acceptor binding cleft and UDP-Gal is in the donor cleft. The departure of UDP is assisted by Mn^2+^ as a Lewis acid and the phenolic group from Tyr-128 as a general acid, leading to a partial interaction of the phenolic oxygen with the anomeric carbon. The acceptor hydroxyl group serves as a nucleophile assisted by the Asp-243 carboxylate as a general base (PDB: 8ZX8). (D) β3GalT5:UDP-Gal:Gb5 glycan ternary structure represents the proposed product formation (PDB: 8ZWW). (E) P-31 signals of UDP, UDP-Gal, β3GalT5 with UDP-Gal, and β3GalT5 with UDP-Gal and Gb4 glycan at the beginning of mixing. Red arrows indicate the P-31 signal of UDP, indicating the enzymatic reaction of UDP-Gal with water as an acceptor is much slower than that with Gb4 glycan as an acceptor. (F) Proposed S_N_2-like mechanisms for β3GalT5-catalyzed hydrolysis of UDP-Gal in the absence of a glycan acceptor to generate galactopyranose. The activated UDP-Gal with oxocarbenium character may collapse to another product through an S_N_1-like mechanism (see the discussion). (G) Proposed S_N_2-like mechanism for reaction with the glycan acceptor.

In this study, we determined three additional ternary structures of β3GalT5. First, in the absence of a glycan acceptor, we observed the exocyclic C1″–O1″ bond breaking in β3GalT5 cocrystallized with UDP-Gal. Second, we observed donor galactose departing from UDP in β3GalT5 cocrystallized with UDP-Gal and soaked with Gb4 glycan. Finally, we investigated β3GalT5 cocrystallized with UDP and soaked with the product Gb5 (SSEA3) glycan. These structures provide insights into the mechanism of the enzymatic reaction. In addition to the structural results, phosphorus-31 NMR spectroscopy shows galactose dissociation at the starting point when the reaction begins to occur (Figures 4E and S7). Thus, we propose that the catalytic mechanism of β3GalT5 involves steps (Figure 4F) diverging from other inverting galactosyltransferases under certain conditions and different from the double displacements proposed for retaining glycosyltransferases.^33,43^

Initially, the glycosidic bond between UDP and the donor galactose is partially broken yet remains within an interacting distance range (approximately 3.34 Å), (Figure 4B). In the enzymatic glycosyl transfer reaction, a metal ion and/or general acid assistance is required to facilitate the cleavage of the exocyclic C1″–O1″ bond. In this case, the departure of the leaving group UDP is assisted by Mn^2+^ as a Lewis acid and the side chain hydroxyl of Tyr128 (2.67 Å) as a general acid (Figure 4B), which initially interacts with both C1″, O1″, and O5″ at distances of 3.31, 3.45, and 3.01 Å, respectively (Figure 4A). However, in the absence of a glycan acceptor, we observed a slow breakdown of UDP-Gal to galactose and another product with electron density such as the oxocarbenium intermediate, presumably a stable structure collapsed from the high-energy oxocarbenium intermediate. Apparently, a water molecule in the active site acts as an acceptor to generate the galactose product (Figure 4F), and this process is assisted by the carboxylate of Asp243 as a general base (Figure S8) to facilitate hydrolysis. The water molecule can also interact with the partially positive charge O5 of the oxocarbenium-like high-energy intermediate and this is consistent with the QM/MM calculations of *Thermus thermophilus* β-glycosidase showing a neighboring water molecule interacting with the oxocarbenium-like intermediate through O5.^44^ Tyr128 and Lys197 further interact with the diphosphate of UDP to facilitate the departure of UDP and the formation of galactopyranose (Figure 4A,B). This background hydrolysis of the donor substrate is often observed in GTs, including β3GalT5, which explains why we observed the dissociation of donor galactose in the absence of an acceptor and at the starting point of the reaction when an acceptor was added.^34,45−47^

In the glycosyl transfer reaction, Asp243 acts as a general base, deprotonating the glycan acceptor 3-OH on the GalNAc (2.32 Å) for a nucleophilic reaction with the anomeric carbon of the galactose (2.71 Å) (Figure 4C), resulting in the formation of a new β-1,3-glycosidic bond between the galactose and the GalNAc (Figure 4D). Our experimental method enabled us to observe these results, likely due to the initial cocrystallization of β3GalT5 with UDP-Gal followed by soaking with the acceptor. This soaking experiment allowed us to capture the breaking of the UDP-Gal bond and the formation of the new glycosylic bond with the acceptor. Collectively, the β3GalT5-catalyzed glycosyl transfer reaction utilizes an S_N_2-type reaction (Figure 4F,G) and more likely a nonconcerted S_N_2-like mechanism. In the enzymatic reaction with weak acceptors like water, the oxocarbenium-like high-energy intermediate may react with water to form galactopyranose through an S_N_2-like mechanism or collapse to a stable product with a similar electron density through an S_N_1-like mechanism. Based on our observations, the enzymatic reaction mechanism appears to be dependent on the reactivity of a bound acceptor. However, for a comprehensive understanding of the reaction with different acceptors, further quantitative analysis and kinetic studies, coupled with improved structural resolution, are necessary to elucidate the details of this dynamic enzymatic reaction at the atomic level.

Even though there are no major conformational changes at the protein level, the side chains of Tyr128, Asp156, Lys197, and Trp198 exhibit significant alterations during the enzymatic reaction (Figures 4A–D and S9–S11). First, the side chain of Tyr128 moves toward the leaving group, UDP, throughout the reaction events, maintaining an approximate distance of 1.09 Å (Figure S9). Second, Asp156 plays a crucial role in transferring the donor galactose to the acceptor. It interacts with the C-3 hydroxyl group on the galactose, with a movement of approximately 0.52 Å toward the acceptor when galactose dissociates from UDP. Upon acceptor binding, this movement increases to 0.84 Å. Interestingly, when the product Gb5 glycan is formed, Asp156 returns to a position close to that observed in the UDP-2FGal bound structure (Figure S10). Third, the side chain of Lys197 stabilizes the UDP leaving group by shifting its interactions from α-phosphate to β-phosphate (Figure 4A–D). Finally, the side chain of Trp198 exhibits dynamic “lid on” and “lid off” conformational change. The angle difference between the initial UDP-2FGal bound structure and the final product bound structure is approximately 33°. The indole lid is turned off when no receptor is bound but is activated by forming a stacking interaction with the nonreducing end of acceptor (Figure S11). In our mutagenesis study, we observed abolished enzymatic activity through mutating the key residues interacting with donor galactose, divalent ion Mn^2+^, acceptor Gb4 glycan, and product Gb5 glycan (Figure S12). These observations provide valuable insights into the dynamic behavior of key residues during the β3GalT5-catalyzed glycosylation process.

### Conformation of Donor Galactose and Coordination Geometry of the Divalent Ion Mn^2+^ in Reaction

**Figure 5:**
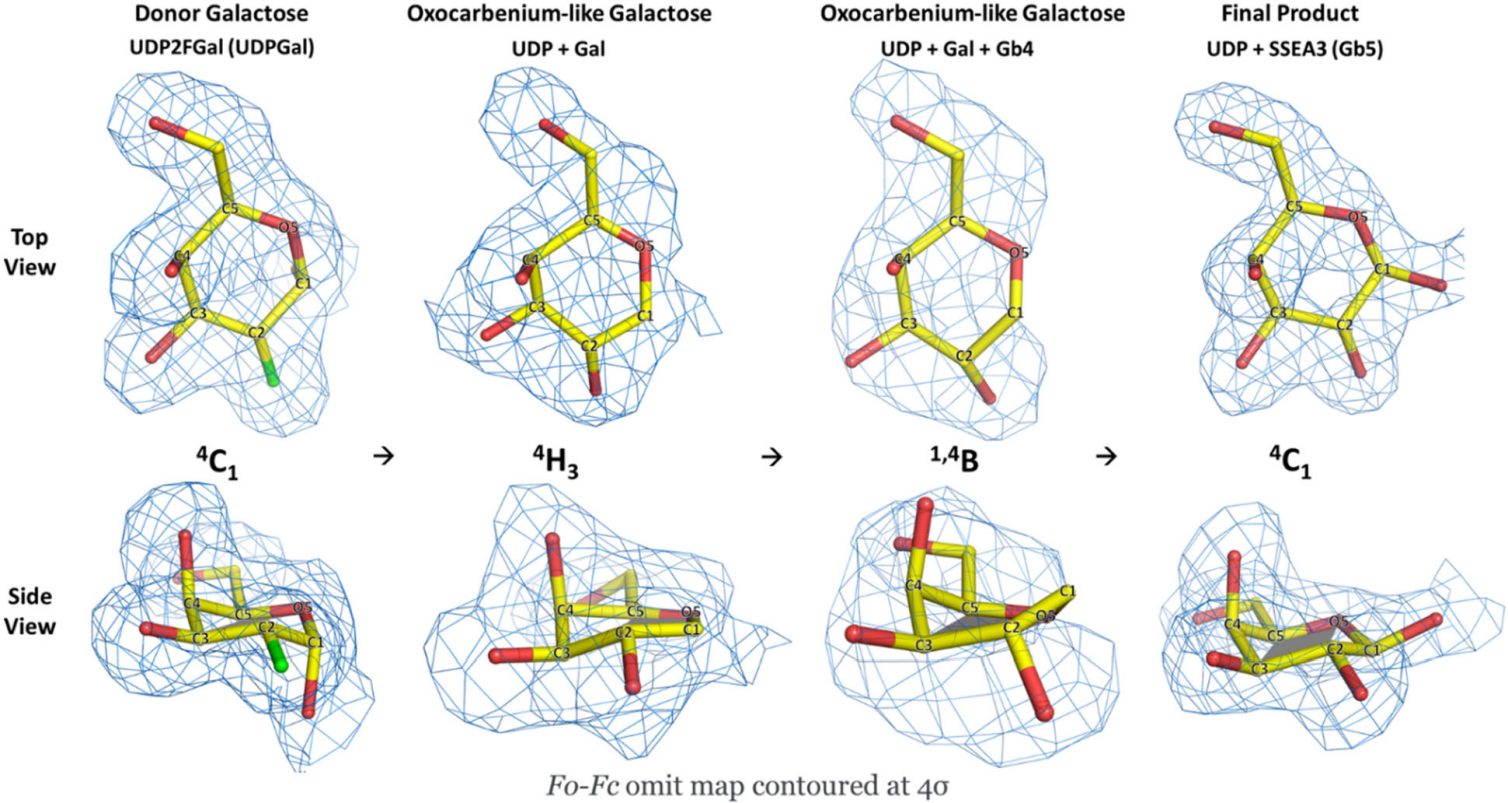
Conformations and electron density maps of donor galactose in enzymatic reaction. The conformations of donor galactose (colored gold) from binding of UDP-Gal to formation of product Gb5 glycan. Side views of galactose show the planar conformation (colored gray). *F*_o_–*F*_c_ polder omit electron-density maps are contoured at 4σ. From left to right, PDB: 8ZWR represents donor galactose from UDP2FGal (UDPGal); PDB: 8ZX9 (UDP + Gal) represents dissociated galactose from the structure of enzyme cocrystallized with UDP-Gal; PDB: 8ZX8 (UDP + Gal + Gb4) represents dissociated galactose from the structure of enzyme cocrystallized with UDP-Gal and soaked with Gb4-glycan; and PDB: 8ZWW (UDP + Gb5) represents the galactose from the final product Gb5-glycan where β3GalT5 is cocrystallized with UDP-Gal and soaked with SSEA3-glycan.

We observed a distortion in the galactopyranose ring upon binding of the donor, potentially indicating the presence of oxocarbenium-like intermediates. The flattened geometry of the donor galactopyranose ring is also consistent with oxocarbenium-like character. Specifically, a partially positive charge (δ+) was developed on the anomeric carbon, which was stabilized by the phenolic oxygen of Tyr128. This portion of the structure must be planar. Consequently, there are eight possible conformers (^4^H_3_, ^3^H_4_, ^2,5^B, B_2,5_, ^4^E, E_4_, ^3^E, and E_3_) for stabilizing the pyranose as an oxocarbenium ion state.^48,49^ The ^1,4^B ring conformation has also been reported in inverting galactosyltranserases^50^ and glycoside hydrolases.^51^ Among these conformers, both the ^4^H_3_ and ^3^H_4_ half-chair conformations are well-supported as the transition state geometry,^52^ and the ^1,4^B, ^2,5^B, and B_2,5_ boat conformations are all identified in the Stoddart diagram.^53^ In the complex structure of β3GalT5 with UDP and dissociated galactose, the galactopyranose ring adopted an ^4^H_3_ half-chair conformation within the catalytic binding domain. However, in the presence of the Gb4-glycan acceptor, the deprotonated 3-OH of the GalNAc acceptor further interacts with the dissociated donor galactose, which might create a potential force to pull the C1 and the galactose pyranose transitioned to the ^1,4^B boat conformation, facilitating bond formation. The weak electron density observed between O5 and the anomeric C1 in the galactopyranose ring may result from constant electron shifts between single and partial double bonds. Subsequently, in complex with the Gb5-glycan product, the galactopyranose ring returned to the ^4^C_1_ chair conformation. Overall, the conformational changes in the galactopyranose ring followed the sequence: ^4^C_1_ → ^4^H_3_ → ^1,4^B → ^4^C_1_ with possibility that there might be other intermediates in between where we yet to capture. Although further mechanistic studies with kinetic studies and computational calculations will support our findings, this study represents the first structure-based observation of the oxocarbenium-like intermediate in an inverted galactosyl transferase-catalyzed reaction (Figure 5).

**Figure 6:**
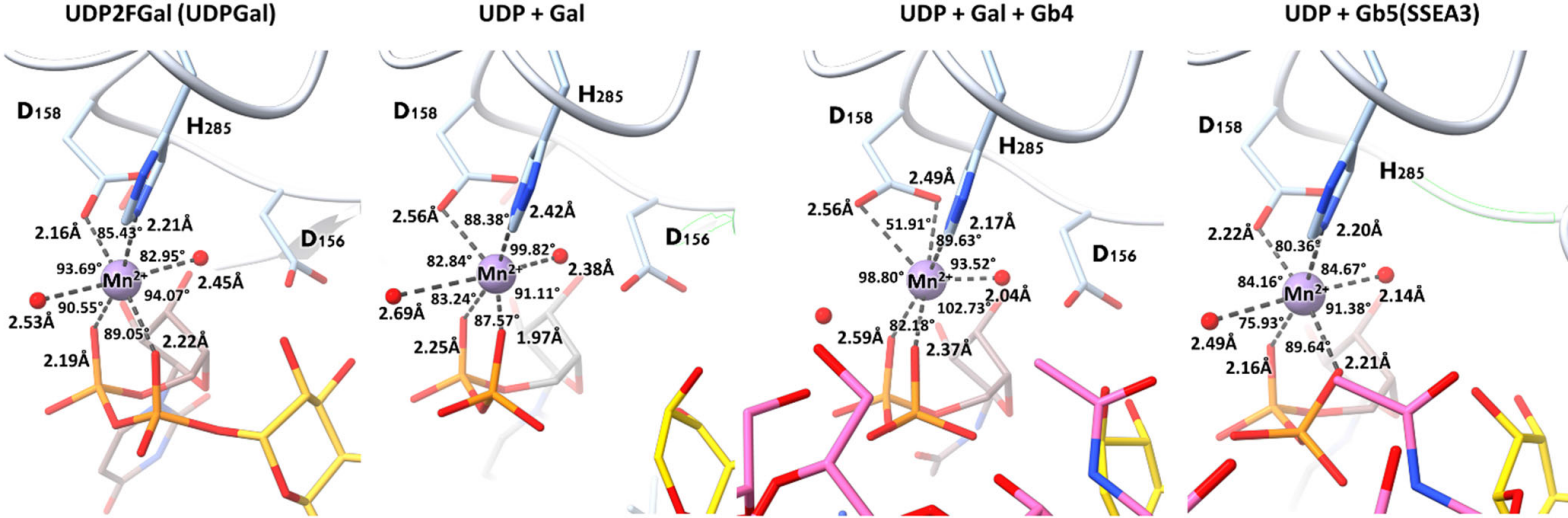
Octahedral geometry of the coordinated divalent ion Mn^2+^. The Mn^2+^(colored purple) octahedral binding partners are Asp158 (from the DXD motif), His 285, diphosphate from UDP, and two water molecules (colored red). In the β3GalT5:UDP:Gal:Gb4 structure, the coordinated partners shift from one water molecule to bidentate Asp 158. The density maps for the four structures are shown in Figure S13 of the Supporting Information.

The donor binding cleft contains a “DXD motif” that consists of an Asp-X-Asp triplet used to coordinate the phosphates of the donor molecule through a divalent cation with an octahedral geometry. In our study, the octahedral geometry surrounding the Mn^2+^ metal is more symmetrical in the UDP-2FGal bound structure with displayed angle ranging from 82.95° to 94.07° and in the Gb5-glycan bound structure with displayed angle ranging from 75.93° to 91.38°. The six bond distances are also more correlated with each other with distances ranging from 2.16 to 2.53 Å for the UDP-2FGal bound structure and 2.14 to 2.49 Å for the Gb5-glycan bound structure. However, when the galactose is in the oxocarbenium-like state, we can observe the distortion with galactose dissociation in the octahedral geometry. In the UDP and Gal bound structure without Gb4-glycan acceptor, the displayed angles range from 82.84° to 99.82° and the bond distances range from 1.97 to 2.69 Å. With Gb4-glycan bound, the octahedral geometry is skewed to nearly trigonal prismatic dimensions with acute bidentate Asp158 coordination employed (displayed angles range from 51.91° to 102.73°) (Figures 6 and S13). The center of the Mn^2+^ coordinated geometry shifts toward the β-phosphate, allows sufficient room for the attack from the acceptor, and facilitates the departure of the leaving group.^54^

## Conclusions

We here provided structural insights into the substrate specificity and possible reaction mechanisms of the β3GalT5-catalyzed reaction. This cancer-related enzyme plays a crucial role in glycolipid biosynthesis and demonstrates intriguing substrate preferences. While it selectively binds to UDP-galactose as the donor substrate, it maintains a broader selectivity for acceptor substrates. Specifically, β3GalT5 catalyzes the transfer of galactose from UDP-Gal to both Gb4 glycan (with GalNAc at the nonreducing end) and Lc3 glycan (with GlcNAc at the nonreducing end) via a β1,3-linkage. In our study of substrate specificity involving disaccharides, β3GalT5 also exhibits robust activities toward Mannose-β1,6-Mannose and GlcNAc-β1,3-GalNAc-α-Thr in addition to Gb4 and Lc3 glycans. We illustrate the key interactions in the glycosylation processes with different acceptor substrates based on the structures. Additionally, we uncover the mechanism of β3GalT5 catalysis by solving the structures of different catalysis steps, including the Michaelis complex, the oxocarbenium-like intermediate, and the reaction products. Based on the structures observed in our study, the β3GalT5-catalyzed glycosyl transfer reaction utilizes an S_N_2-like mechanism, where the donor substrate UDP-Gal is activated by Mn^2+^ and Tyr-128 to facilitate the departure of UDP and the acceptor hydroxyl group acts as a nucleophile to react with the anomeric carbon of UDP-Gal under the assistance of Asp243 carboxylate as a general base to generate a new glycosidic bond in β1,3-linkage. However, in the enzymatic reaction without a glycan acceptor, our structural studies indicate that UDP-Gal is activated and reacted with water as an acceptor through an S_N_2-like mechanism to form the galactopyranose product. In addition, the activated UDP-Gal with an oxocarbenium-like structure may collapse through an S_N_1-like mechanism to form a product with similar electron density to the oxocarbenium intermediate of galactose, likely the enol form of galactal (Figure S14). These observations suggest that the mechanism of the enzymatic reaction may change, depending on the reactivity of the bound acceptor, and could range from S_N_2-like for reaction with good acceptors to S_N_1-like mechanism for weak acceptors. It is noted that the proposed mechanisms are based on the structures obtained from our experiments and may not precisely reflect the reactions in the solution. Nevertheless, the broad-spectrum acceptor substrate specificity and the thorough investigation into the structure, function, and mechanism of β3GalT5 not only enhance our understanding of its potential in glycan synthesis but also illuminate its crucial role in cancer biology. The findings offer new opportunities for synthetic glycobiology and drug design, specifically targeting β3GalT5 in diseases.

## Supporting Information Available

Synthetic and experimental procedures, characterization and structural analyses, and crystallographic information (PDF)

## Accession Codes

Atomic coordinates and structural factors for the reported 8 crystal structures have been deposited in the Protein Data Bank under the accession number (8ZWR, 8ZWP, 8ZWW, 8ZWY, 8ZX2, 8ZX3, 8ZX8, and 8ZX9). Other data are available from the corresponding author upon reasonable request.
