# Supplementary material for: Structure-Based Mechanism and Specificity of Human Galactosyltransferase β3GalT5
Source: J Am Chem Soc. 2025 Mar 25;147(13):10875–85. doi: 10.1021/jacs.4c11724 (PMC11969544; doi:10.1021/jacs.4c11724)
Supplement: Supplementary file 1 — ja4c11724_si_001.pdf [file ja4c11724_si_001.pdf]

Supporting Information

Structure-Based Mechanism and Specificity of Human Galactosyltransferase β3GalT5

Jennifer M. Lo,[1,2,3‡] Chih-Chuan Kung,[1‡] Ting-Jen Rachel Cheng,[1] Chih-Huey Wong*[1,4] and Che Ma*[1,2]

[1] Genomics Research Center, Academia Sinica, Taipei 115, Taiwan.
[2] Chemical Biology and Molecular Biophysics Program, Taiwan International Graduate Program, Academia Sinica, Taipei 115, Taiwan.
[3] Department of Chemistry, National Tsing Hua University, Hsinchu 300, Taiwan.
[4] Department of Chemistry, Scripps Research, California 92037, USA.

‡ These authors contributed equally
* Correspondence: cma@gate.sinica.edu.tw or wong@scripps.edu

Table of contents

Materials and Methods S2-9

Supplementary Figures S10

Figure S1. Acceptor substrate specificity catalyzed by β3GalT5. .... S10

Figure S2. Protein preparation and X-ray structure analysis of β3GalT5 .... S11

Figure S3. Structural homologues of β3GalT5 .... S12

Figure S4. Donor substrate specificity catalyzed by β3GalT5 .... S13

Figure S5. Structural and kinetic analysis of β3GalT5 with variant acceptor substrates S14

Figure S6. Electron density maps of the acceptor substrates S15

Figure S7. UDP-Galactose and UDP Phosphorus-31 signal by NMR spectroscopy S16

Figure S8. Electron density maps of the water network in β3GalT5:UDP:Gal (PDB: 8ZX9) structure S17

Figure S9. Tyrosine 128 conformational changes during reaction S18

Figure S10. Aspartic acid 156 conformational changes during reaction S19

Figure S11. Tryptophan 198 conformational changes during reaction S20

Figure S12. Assessment of β3GalT5 point mutants S21

Figure S13. Electron density maps of the octahedral geometry of coordinated divalent ion Mn^{2+} S22

Figure S14. Electron density maps of the oxocarbenium-like galactose and enol pyranose glycal S23

Table S1. X-ray crystallographic data collection and structure refinement statistics (part 1). S24

Reference S26

Materials and Methods

Cloning and protein purification

Protein concentration was estimated by the Bradford assay using BSA as a standard. The expression construct of human β3GalT5 (UniProt Q9Y2C3) luminal domain (Phe31-Pro308) was designed with an N-terminal gp67 secretion signal peptide and a TEV cleavable C-terminal 2xStrep and 8xHis tag. The whole segment was cloned into pBAC1 vector (Novagen) with the traditional cloning method with restriction enzymes (New England Biolabs). The resulting plasmid was co-transfected with flashBACTM ULTRA (OXFORD Expression Technologies) into sf9 cells using CellfectinTM II (Gibco) and high-titer recombinant baculovirus stock was then generated according to manufacturer's instructions. Sf9 cells with a density of 2.5 x 106 cells/ml in Sf-900™ II serum-free medium (Gibco) were infected with P3 baculovirus at a multiplicity of infection of 5. Spent medium was harvested 96 h post infection by centrifugation at 2000g for 15 min.

The collected supernatant was flowed through open column with Ni-NTA resin pre-equilibrated with 20mM HEPES-NaOH pH 7.4, 300mM NaCl (buffer A). Impurities were removed by washing the resin with buffer A supplemented with 30mM imidazole for 20 column volume (CV) followed by buffer A supplemented with 50mM imidazole for 1.5 CV. β5GalT5 was eluted with buffer A containing 200mM imidazole. The elution fractions were buffer exchanged to buffer A using HiPrep 26/10 Desalting column (cytiva), then concentrated to 0.5mg/ml protein concentration using Amicon ultra centrifugal 30kDa molecular weight cutoff filter (Merck) for tag removal by TEV protease. Preliminarily purified protein was concentrated and subject to size exclusion chromatography with Superdex 200 10/300 column (cytiva) in buffer 20mM HEPES-NaOH pH 7.4, 50mM NaCl. To calculate the molecular weight standard curve, the following proteins (Bio-Rad Laboratories, Inc) were used: bovine thyroglobulin (670kDa), bovine γ-globulin (158kDa), chicken ovalbumin (44kDa), horse myoglobin (17kDa), and vitamin B12 (1.35kDa).

Site-directed mutagenesis

Site-directed point mutation mutants T64A, S66A, Q69A, D126A, Y128A, K134A, K154A, D156N, S157A, D158N, K185A, E188A, K197A, W198A, S215A, E242A, D243N, H285A on the pBAC1 (Novagen) plasmid containing human β3GalT5 luminal domain (Phe31-Pro308) described above were made using Q5 Site-Directed Mutagenesis Kit (New England Biolabs). The basic procedure started with the template (wildtype β3GalT5) and two synthetic

oligonucleotide primers containing the desired mutation. The mutagenesis primers were shown in the Primer Table and were synthesized by Genomics (Taipei, Taiwan). The primers were extended during temperature cycling using NEBuilder HiFi DNA polymerase (New England Biolabs). After temperature cycling, the product was treated with DpnI, which digested the parental DNA template, but not the synthesized DNA containing the mutation. The nicked vector DNA incorporating the desired mutation was then transformed into competent cells and the mutants obtained confirmed by DNA sequencing. Protein expression and purification were the same as wild type β3GalT5 described above. The enzyme activities were measured in relative activity toward wild type β3GalT5 using UDP-Glo assay (Promega).

Table S1. Primers used for preparing β3GalT5 mutants

T64A	
Forward	5'-GCTGCTGGTCGCGTCCTCCCATA-3'
Reverse	5'-TATGGGAGGACGCGACCAGCAGC-3'
S66A	
Forward	5'-GGTCACCTCCGCGCATAAGCAGC-3'
Reverse	5'-GCTGCTTATGCGCGGAGGTGACC-3'
Q69A	
Forward	5’-CTCCCATAAGGCGCTCGCCGAGC-3’
Reverse	5’-GCTCGGCGAGCGCCTTATGGGAG-3’
D126A	
Forward	5’-GGACTTCCTCGCGGTGTACTACAAC-3’
Reverse	5’-GTTGTAGTACACCGCGAGGAAGTCC-3’
Y128A	
Forward	5'-CCTCGACGTGGCGTACAACCTGACC-3'
Reverse	5'-GGTCAGGTTGTACGCCACGTCGAGG-3'
K134A	
Forward	5’-CCTGACCCTTGCGACTATGATGGG-3’
Reverse	5’-CCCATCATAGTCGCAAGGGTCAGG-3’

K154A	
Forward	5’-CTTCGTGATGGCGACCGACTCCG-3’
Reverse	5’-CGGAGTCGGTCGCCATCACGAAG-3’
D156N	
Forward	5’-GATGAAAACCAACTCCGACATGTTTATC-3’
Reverse	5’-GATAAACATGTCGGAGTTGGTTTTCATC-3’
S157A	
Forward	5’-GAAAACCGACGCGGACATGTTTATC-3’
Reverse	5’-GATAAACATGTCCGCGTCGGTTTTC-3’
D158N	
Forward	5’-AACCGACTCCAACATGTTTATCAAC-3’
Reverse	5’-GTTGATAAACATGTTGGAGTCGGTT-3’
K185A	
Forward	5’-TGGCTTTCTTGCGCTGAATGAGTTC-3’
Reverse	5’-GAACTCATTCAGCGCAAGAAAGCCA-3’
E188A	
Forward	5’-TAAGCTGAATGCGTTCCCGATCC-3’
Reverse	5’-GGATCGGGAACGCATTCAGCTTA-3’
K197A	
Forward	5’-GCCATTCTCAGCGTGGTTCGTGTCC-3’
Reverse	5’-GGACACGAACCACGCTGAGAATGGC-3’
W198A	
Forward	5’-ATTCTCAAAGGCGTTCGTGTCCAAAAGCGAATACC-3’
Reverse	5’-GGTATTCGCTTTTGGACACGAACGCCTTTGAGAAT-3’
S215A	
Forward	5’-TCCATTCTGCGCGGGCACTGGAT-3’

Reverse	5'-ATCCAGTGCCCGCGCAGAATGGA-3'
E242A	
Forward	5'-CATTAAGCTCGCGGACGTGTTCG-3'
Reverse	5'-CGAACACGTCCGCGAGCTTAATG-3'
D243N	
Forward	5'-TAAGCTCGAAAACGTGTTCGTGG-3'
Reverse	5'-CCACGAACACGTTTTCGAGCTTA-3'
H285A	
Forward	5'-CGTCGCCTGCGCGTTCATCAAGCCCC-3'
Reverse	5'-GGGGCTTGATGAACGCGCAGGCGACG-3'

Crystallization and data collection

β5GalT5 purified from gel filtration was concentrated to 60mg/ml and added 5mM MnCl2. Initial crystallization screening was set up with 15.9mM UDP-α-D-galactose, Disodium Salt (UDPGal; Millipore) or 15.9mM UDP-2-deoxy-2-fluoro-D-galactose (UDP2FGal; BOC Sciences) using the hanging-drop vapor diffusion method with commercial screening kits at 4°C. Diffraction-quality crystals appeared in condition 100mM BIS-TRIS Propane pH8.5, 200mM Potassium Sodium Tartrate, 20% w/v PEG3350 with rod shape cluster. Optimization of crystals was carried out with hanging-drop vapor diffusion method in 24-well trays (Hampton Research). Crystals were generally formed after a 7day incubation. Before X-ray diffraction data collection, crystals were transferred to the same mother liquor containing 20% glycerol as a cryoprotectant. To obtain β3GalT5_UDPGal with different acceptor substrates, β3GalT5_UDPGal crystals were soaked in soaking solution containing 15.9mM Gb4, Gb5/SSEA3, GlcNac-β1,3-Gal-OMe of Lc3, Mannose-β1,6-Mannose, or GlcNAc-β1,3-GalNAc-α-Thr of Core3 O-linked glycan in 4°C and harvested with 15 minutes interval from 1 minute to 3 hours. The best resolution datasets were used. All crystal diffraction results were collected on an ADSC Quantum-315 or MX300HE CCD detectors in an operating temperature of 100 K at beamline 13B1, 13C1, 15A1, 05A at the National Synchrotron Radiation Research Center (NSRRC) (Taiwan). Data were indexed and scaled with the HKL2000 package[1].

Table S2. X-ray crystallization and harvesting conditions for all presented structures, including specific conditions for co-crystallization and/or substrate soaking, as well as specific harvesting temperatures and time intervals.

	PDB code	Crystallization conditions
β3GalT5:UDP-2FGal	8ZWR	60mg/ml (1.59mM) of β3GalT5 was co-crystallized with 15.9mM of UDP-2FGal (BOC Sciences).
β3GalT5:UDP:Gal *	8ZX9	60mg/ml (1.59mM) of β3GalT5 was co-crystallized with 15.9mM of UDP-α-D-galactose, disodium salt (Millipore).
β3GalT5:UDP:Gal:Gb4 *	8ZX8	60mg/ml (1.59mM) of β3GalT5 was co-crystallized with 15.9mM of UDP-α-D-galactose, disodium salt (Millipore). The crystals were transferred to mother liquid containing 15.9mM of Gb4 glycan part (ELICITYL). Crystals were incubated in 4°C and harvested with 15 minutes interval from 1 minute to 3 hours. The best resolution datasets were used. The best resolution dataset was used. The dataset used here is from 15mintues soaking time for 8ZX8 and 1 hour soaking time for 8ZWP.
β3GalT5:UDP:Gb4	8ZWP	
β3GalT5:UDP:Gb5/SSEA3	8ZWW	60mg/ml (1.59mM) of β3GalT5 was co-crystallized with 15.9mM of UDP-α-D-galactose, disodium salt (Millipore). The crystals were transferred to mother liquid containing 15.9mM of Gb5, so called SSEA3, glycan part. Crystals were incubated in 4°C and harvested with 15 minutes interval from 1 minute to 3 hours. The best resolution dataset was used. The dataset used here is from 1 hour and 15 minutes soaking time.
β3GalT5:UDP:GlcNac-β1,3-Gal-OMe	8ZWY	60mg/ml (1.59mM) of β3GalT5 was co-crystallized with 15.9mM of UDP-α-D-galactose, disodium salt (Millipore). The crystals were transferred to mother liquid containing 15.9mM of GlcNac-β1,3-Gal-OMe (Biosynth). Crystals were incubated in 4°C and harvested with 15 minutes interval from 1 minute to 3 hours. The best resolution dataset was used. The dataset used here is from 2 hours soaking time.

β3GalT5:UDP: GlcNac-β1,3-GalNAc-α-Thr	8ZX3	60mg/ml (1.59mM) of β3GalT5 was co-crystallized with 15.9mM of UDP-α-D-galactose, disodium salt (Millipore). The crystals were transferred to mother liquid containing 15.9mM of GlcNac-β1,3-GalNAc-α-Thr (Biosynth). Crystals were incubated in 4°C and harvested with 15 minutes interval from 1 minute to 3 hours. The best resolution dataset was used. The dataset used here is from 45 minutes soaking time
β3GalT5:UDP: Man-β1,6-Man *	8ZX2	60mg/ml (1.59mM) of β3GalT5 was co-crystallized with 15.9mM of UDP-α-D-galactose, disodium salt (Millipore). The crystals were transferred to mother liquid containing 15.9mM of Man-β1,6-Man (Biosynth). Crystals were incubated in 4°C and harvested with 15 minutes interval from 1 minute to 3 hours. The best resolution dataset was used. The dataset used here is from 3 hour soaking time

* It was observed that most of the obtained crystal structures exhibited very weak galactose density. The three datasets with the best resolution were selected: β3GalT5:UDP:Gal (PDB: 8ZX9), β3GalT5:UDP:Gal:Gb4 (PDB: 8ZX8), and β3GalT5:UDP:Gal:Man-β1,6-Man (8ZX2).

Structure determination and refinement

The phase was solved by sulfur-single-wavelength anomalous dispersion from β3GalT5 co-crystallized with UDP-Gal. Collected data was indexed, integrated, and scaled using HKL2000.[1] Autosol[2] in Phenix suite was used for locating the 20 sulfur atoms, phasing, density modification, and model building. The model was then used for molecular replacement (MR) in Phenix.phaser[3] and iterative manual adjustment in COOT[4] and refinement of the model using Phenix.refine[5] were carried out to achieve the final structures. Polypeptide models were built with the COOT[4] and further refined with REFMAC[6] and PHENIX[5]. Detailed refinement statistics are given in **Table S1**. Figures were generate using PyMOL (http://www.pymol.org)[7] and ChimeraX.[8]

Enzymatic reactions

To determine the kinetic mechanism of β3GalT5 **(Figure S5D)**, enzyme activity was evaluated using the UDP-Glo assay (Promega), which detects UDP production from the enzymatic reaction with UDP-Gal in the presence or absence of a glycan acceptor. In the absence of glycan

acceptors, water would act as acceptor to hydrolyze UDP-Gal to UDP and Gal. Following the manufacturer's protocol, a standard curve was generated with UDP concentrations ranging from 0–25 μM, establishing a linear detection range where luminescence is directly proportional to UDP concentration. For assessing the kinetics of the Gb4 glycan part and Man-β1,6-Man, a reaction mixture was prepared containing 20 mM HEPES (pH 7.4), 0.1 mM $MnCl_2$, 10 mM UDP-galactose (Promega), and nine glycan substrate concentrations (3, 2, 1.5, 1, 0.75, 0.5, 0.25, 0.125, and 0 mM) with 0.5 μM β3GalT5 enzyme. Similarly, for GlcNAc-β1,3-Gal-OMe kinetics, nine glycan substrate concentrations (1, 0.75, 0.5, 0.375, 0.25, 0.188, 0.125, 0.063 mM) were combined with 0.1 μM β3GalT5 in a solution containing 20 mM HEPES (pH 7.4), 0.1 mM $MnCl_2$, and 5 mM UDP-galactose. For GlcNAc-β1,3-GalNAc-Thr kinetics, the same concentration series was used with 0.5 μM β3GalT5 in 20 mM HEPES (pH 7.4), 0.1 mM $MnCl_2$, and 5 mM UDP-galactose. All reactions were incubated at 37°C for 0-2 minutes before quenching, and luminescence was measured using a CLARIOstar microplate reader (BMG Labtech). Data were analyzed with GraphPad Prism 10, fitting to the Michaelis-Menten model, and results are reported as the mean of three biological replicates ± SEM.

To evaluate the relative activity of different acceptor substrates to Gb4 **(Figures S1 and S5C)** or of UDP sugar donor substrates to UDP-galactose **(Figure S4)**, an enzymatic solution was prepared containing 20 mM HEPES (pH 7.4), 0.1 mM $MnCl_2$, 0.1 mM UDP-galactose, and 5 mM different acceptor substrates or 0.1 mM different UDP-sugars along with 5 mM of Gb4 glycan. Recombinant β3GalT5 (5 μM) was then added simultaneously to ensure all compared samples had the same starting point. Relative activity was calculated by dividing each sample reading by the Gb4 glycan sample reading or UDP-galactose sample reading and multiplying by 100%.

Similarly, to assess the enzymatic activity of β3GalT5 mutants compared to wild-type β3GalT5 **(Figure S12)**, an enzymatic solution containing 20 mM HEPES (pH 7.4), 0.1 mM $MnCl_2$, 0.1 mM UDP-galactose, and 5 mM of Gb4 glycan was mixed, and 0.5 μM of either β3GalT5 mutants or wild-type β3GalT5 was added simultaneously to standardize starting conditions. The fold change was calculated by dividing the reading of each mutant by that of the wild-type.

NMR 31P spectra measurements

The NMR experiments were conducted at 298 K on a Bruker Avance 500 MHz NMR spectrometer equipped with a 5 mm triple resonance cryoprobe with a Z-axis gradient. 31P chemical shifts were externally referenced to 0 ppm phosphorus resonance of H3PO4 in D2O. NMR data were collected and processed using Topspin 2.1 software (Bruker, Germany) and further analyzed by Sparky version 4.1.3 (Goddard and Kneller).

31P NMR spectra were recorded at different time points: 0 hours, 4 hours, 22 hours, and 5 days. The samples used in these experiments contained 0.16 mM β3GalT5 with 0.5 mM MnCl2 in 500 μL of approximately 100% D2O buffer, which included 20 mM HEPES (pH 7.4) and 50 mM NaCl. 1.59mM UDP-Gal and Gb4 were added right before loading onto Bruker Avance 500 MHz.

The 31P NMR (500 MHz) spectra were obtained for the following samples: 1. UDP only (1.59mM). 2. UDP-Gal only (1.59mM). 3. β3GalT5 (0.16mM) with $MnCl^2$ (0.5mM) and UDP-Gal (1.59mM). 4. β3GalT5 (0.16mM). All samples were prepared in 500 μL of approximately 100% D2O buffer, containing 20 mM HEPES (pH 7.4) and 50 mM NaCl.

Supplementary Figures

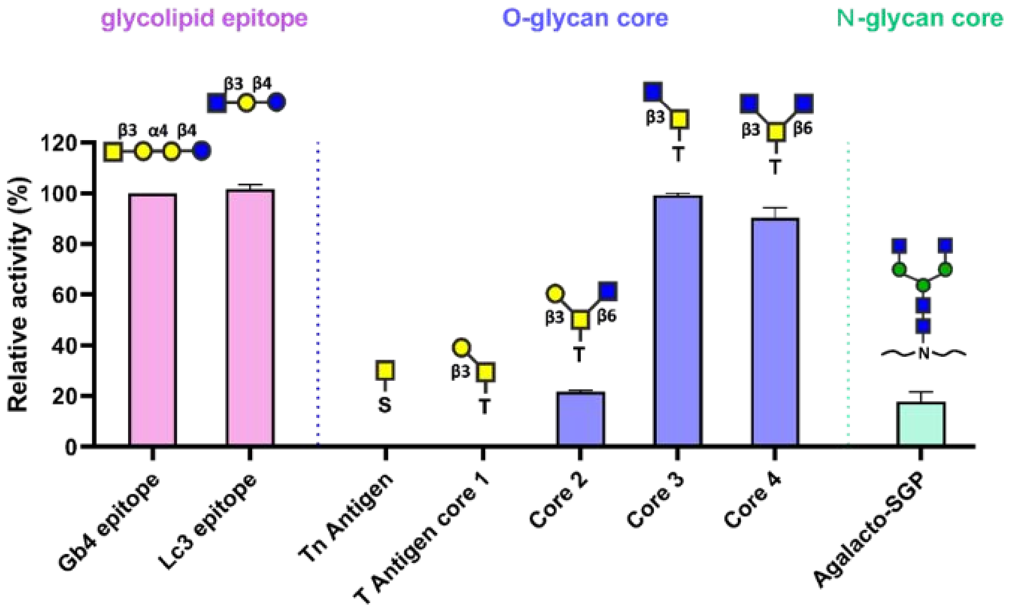

Figure S1. Acceptor substrate specificity catalyzed by β3GalT5. Acceptor substrates specificity is shown with relative activity to Gb4 glycan. Glycolipid epitopes are represented in pink bar, O-glycan cores are represented in blue bar, and N-glycan core is represented in green bar. Table next of it shows relative activity in percentage. To evaluate the relative activity of different acceptor substrates to Gb4 glycan, an enzymatic solution is prepared containing 20 mM HEPES (pH 7.4), 0.1 mM $MnCl_2$, 0.1 mM UDP-galactose with 5 mM acceptor substrates and 5μM β3GalT5 using UDP-Glo assay. Relative activity is calculated by dividing each sample reading by the Gb4 glycan sample reading and multiplying by 100%. β3GalT5 shows preference in Gb4, Lc3, Core3 and Core 4 O-glycan core as acceptor substrate.

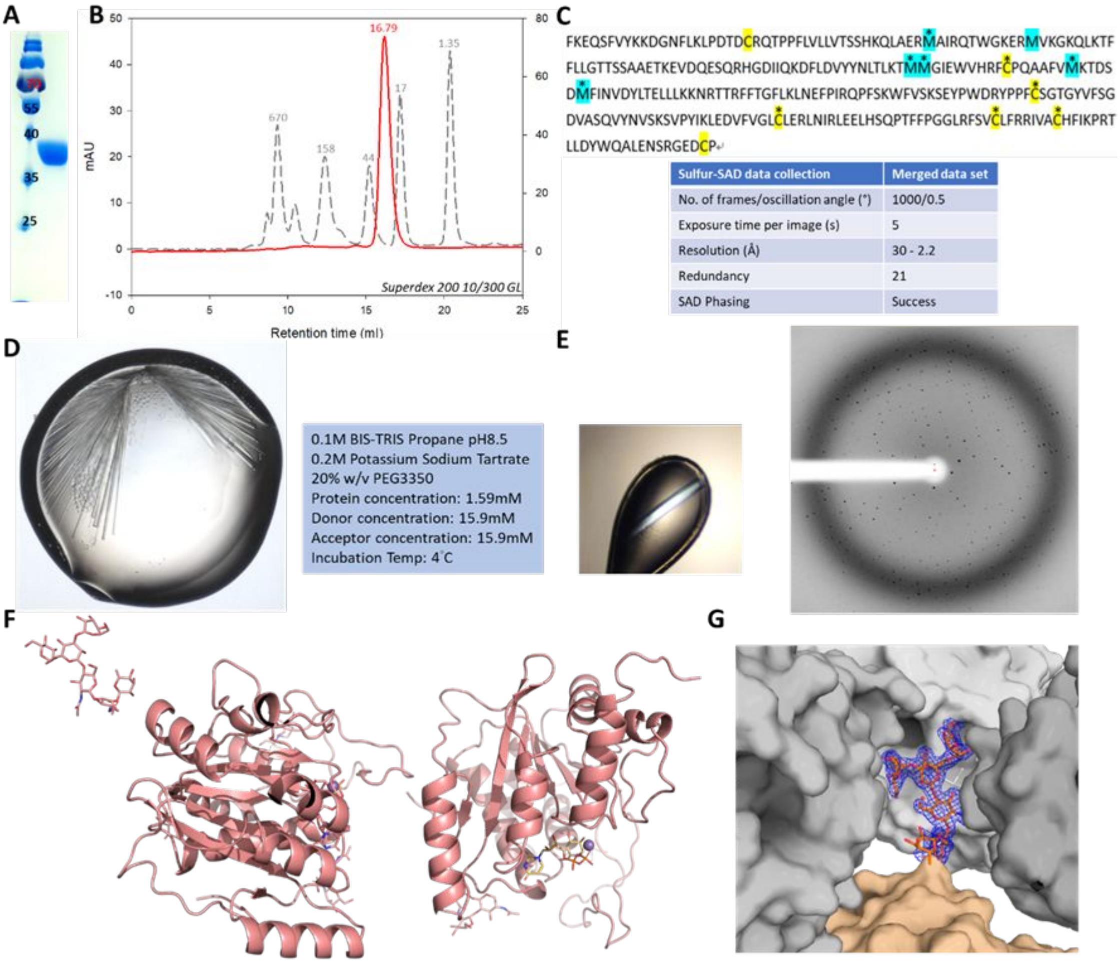

Figure S2. Protein preparation and X-ray structure analysis of β3GalT5. A. SDS-PAGE of β3GalT5 shows a molecular weight around 37kDa. **B.** Gel-filtration of a homogenous β3GalT5. It indicates β3GalT5 is a monomer. **C.** X-ray structure determination by sulfur single-wavelength anomalous dispersion phasing. **D.** β3GalT5 co-crystallized with UDP-galactose forming an angel wing-like needle cluster. Text box shows crystallizing condition with ultra-high protein concentration (60mg/ml). **E.** Diffraction pattern of a single crystal. **F.** An asymmetric unit of β3GalT5 crystals contains two polypeptide chains. **G.** Paucimannose N-glycosylation site 174 stabilized the crystal packing with adjacent molecules.

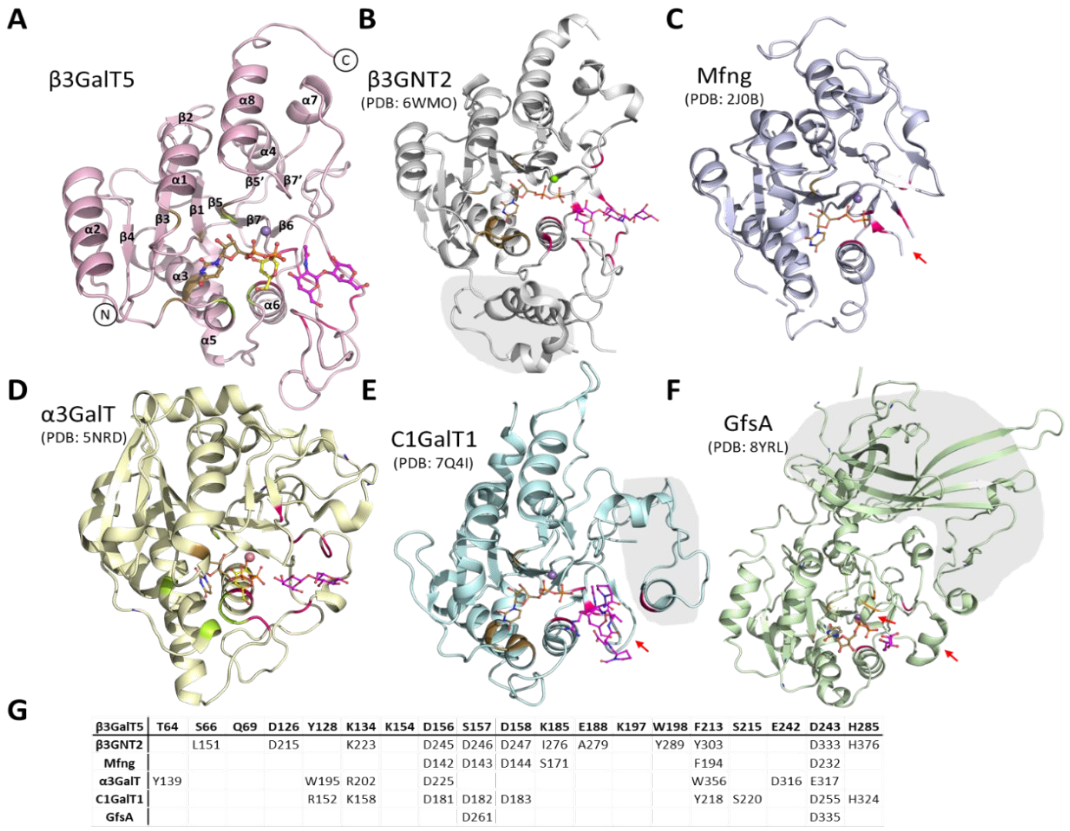

G

β3GalT5	T64	S66	Q69	D126	Y128	K134	K154	D156	S157	D158	K185	E188	K197	W198	F213	S215	E242	D243	H285
β3GNT2		L151		D215		K223		D245	D246	D247	I276	A279		Y289	Y303			D333	H376
Mfng								D142	D143	D144	S171				F194			D232	
α3GalT	Y139				W195	R202		D225							W356		D316	E317	
C1GalT1					R152	K158		D181	D182	D183					Y218	S220		D255	H324
GfsA									D261									D335	

Figure S3. Structural homologues of β3GalT5. The UDP nucleotide is shown in brown, the donor sugar in yellow, and the acceptor in magenta. Brown-colored cartoons represent residues interacting with the UDP moiety. Lime-colored cartoons represent residues interacting with the donor sugar. Hot pink-colored cartoons represent residues interacting with the acceptor. Ligands and interacting sites from all structures follow the same color scheme. **A.** β3GalT5:UDP:Gal:Gb4 structure in the presence of the metal cofactor Mn^{2+}. Only the two terminal end disaccharides of Gb4 are shown. **B.** B3GNT2 (PDB: 6WMO) structure with UDP and acceptor lacto-N-neo-tetraose (LNnT) in the presence of the metal cofactor Mg^{2+}. The grey background represents the additional N-terminal motif (α1 to α3). **C.** Mfng (PDB: 2J0B) structure with UDP in the presence of the metal cofactor Mn^{2+}. The red arrow indicates the missing disordered "long loop". **D.** α3GalT (PDB: 5NRD) structure with UDP-Gal and acceptor lactose (LAT) in the presence of the metal cofactor Co^{2+}. **E.** *Dm*C1GalT1 (PDB: 7Q4I) structure with UDP and glycopeptides APDTR*P with GalNAc as acceptor in the presence of the metal cofactor Mn^{2+}. The grey background represents the additional C-terminal motif (α7 to α9), and the red arrow indicates the shorter "long loop" region. **F.** *Af*GfsA (PDB: 8YRL) structure with UDP and galactose in the presence of Mn^{2+}. The grey background represents the additional motif composed of β-sheets, and the red arrows indicate the replacement of the "long loop" with two α-helix. **G.** Relative interacting residues among different GTs. Only the interacting residues mentioned in the manuscripts are shown.

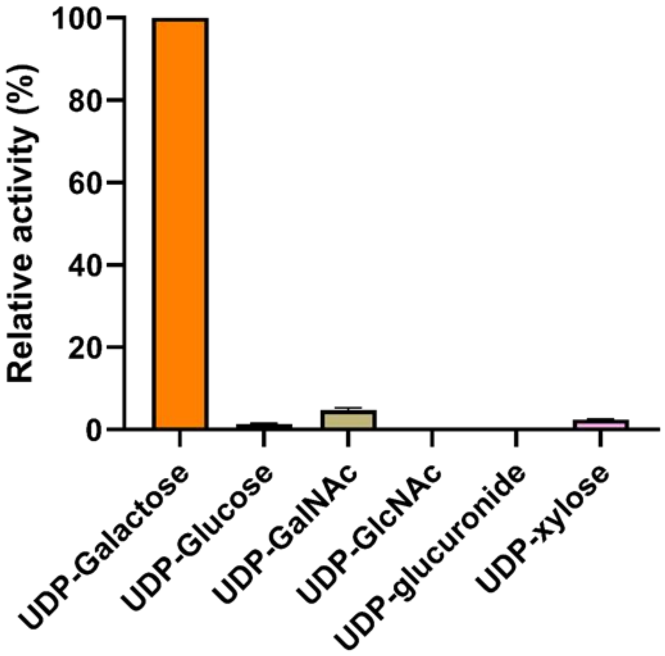

Figure S4. Donor substrate specificity catalyzed by β3GalT5. Nucleotide sugar donor substrates specificity is shown with relative activity to UDP-galactose and bars are colored in orange. To evaluate the relative activity of different UDP sugar donor substrates to UDP-galactose, an enzymatic solution is prepared containing 20 mM HEPES (pH 7.4), 0.1 mM $MnCl_2$, 0.1 mM UDP-galactose or other UDP sugars along with 5 mM of Gb4 glycan and 5μM of β3GalT5 using UDP-Glo assay. Relative activity was calculated by dividing each sample reading by the UDP-galactose sample reading and multiplying by 100%. β3GalT5 selectively binds to UDP-galactose as the donor substrate.

A

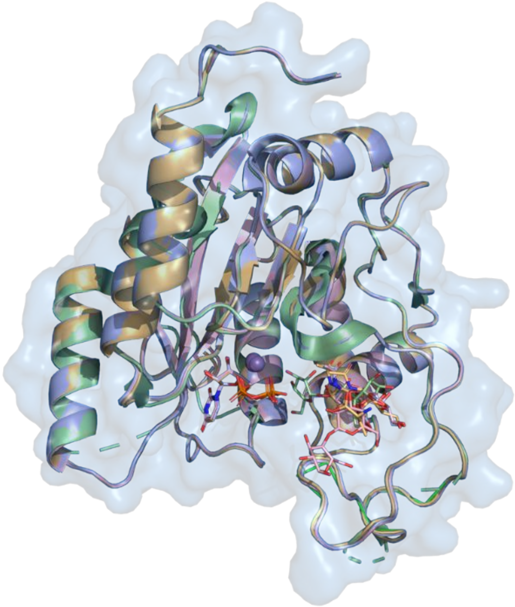

Compared to GalNAc-β1,3-Gal (Gb4)	r.m.s.d (Å)	Cα (atoms)
GlcNAc-β1,3-Gal-OMe (Lc3)	0.10	234
GlcNAc-β1,3-GalNAc-α-Thr	0.14	229
Man-β1,6-Man	0.15	238

B

Substrate	Symbol	Km(mM)	kcat(min^{-1})	kcat/Km($min^{-1}mM^{-1}$)
Gb4 glycan	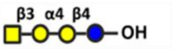	2.0±0.3	117.0±8.5	58.6±31.9
GlcNAc-β1,3-Gal-OMe	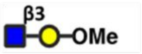	0.5±0.04	228.3±8.3	461.1±227.0
GlcNAc-β1,3-GalNAc-Thr	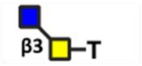	0.75±0.1	83.8±6.8	111.1±52.7
Man-β1,6-Man	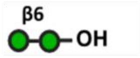	2.5±0.4	37.9±3.5	15.3±9.1

Figure S5. Structural and kinetic analysis of β3GalT5 with various acceptor substrates. A. Superimposition of β3GalT5:UDP-Galactose with glycan part of Gb4 (in pink), disaccharide GlcNac-β1,3-Gal-OMe of Lc3 (in purple), disaccharide GlcNAc-β1,3-GalNAc-α-Thr (in orange), and disaccharide Man-β1,6-Man (in green). The table shows the r.m.s.d (Å) and Cα (atoms), suggesting that there are no significant conformational changes at protein level. **B.** Kinetic parameters of β3GalT5 reacting with various acceptor substrates.

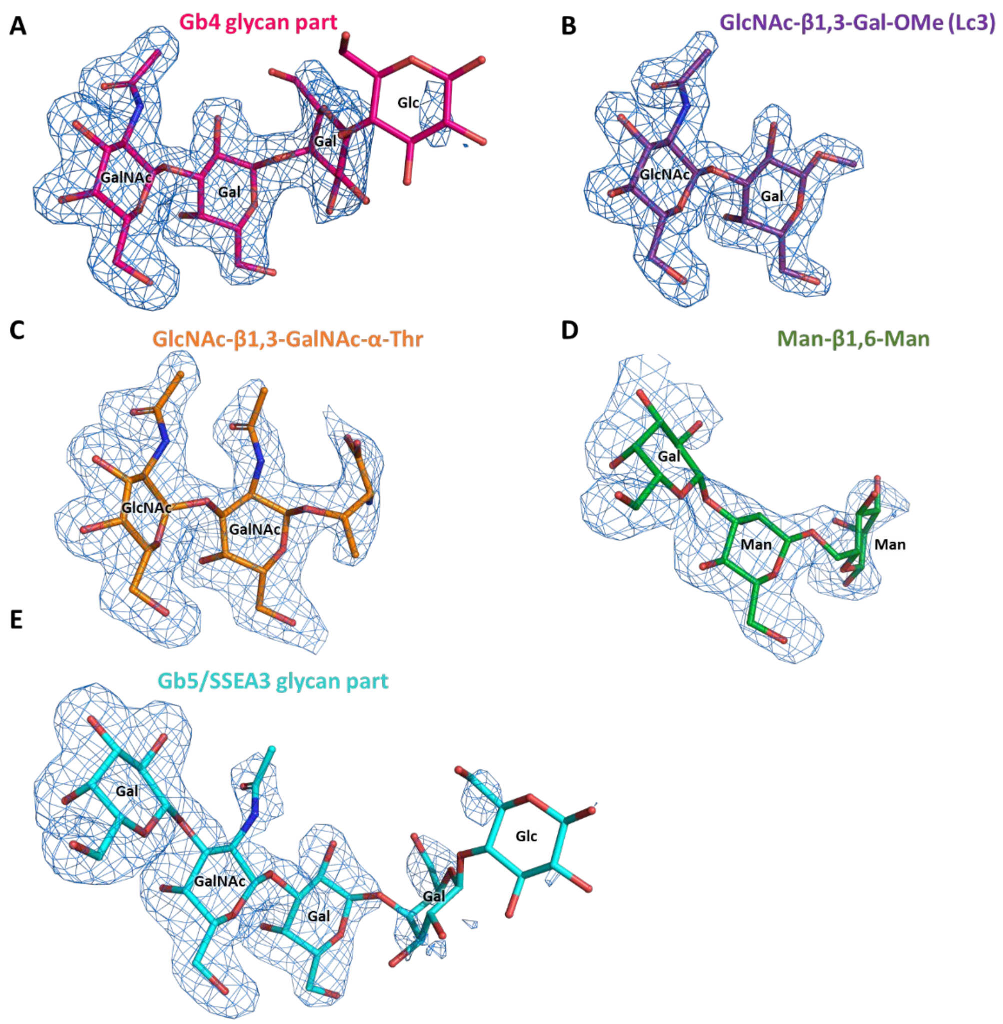

Figure S6. Electron density maps of the acceptor substrates. The structures are obtained by soaking of acceptors (Gb4 glycan, GlcNac-β1,3-Gal-OMe, GlcNAc-β1,3-GalNAc-α-Thr, or Man-β1,6-Man) or product (Gb5/SSEA3 glycan) with the β3GalT5 co-crystallized with UDP-Gal. The Polder omit maps are generated using PyMOL software, and F_o-F_c electron-density maps were contoured at 3σ. **A.** The omit map for Gb4 glycan acceptor (colored pink). **B.** The omit map for GlcNac-β1,3-Gal-OMe acceptor (colored purple) of Lc3. **C.** The omit map for GlcNAc-β1,3-GalNAc-α-Thr acceptor (colored orange) of core 3 O-linked glycan. **D.** The omit map for Gal-β1,3-Man-β1,6-Man (colored green). Although the structure is obtained by soaking of Man-β1,6-Man acceptor, the density map shows clearly a product-like substrate, Gal-β1,3-Man-β1,6-Man, probably due to a longer soaking time (3 hours). **E.** The omit map for Gb5/SSEA3 product (colored cyan). All the above structures are in the same orientation.

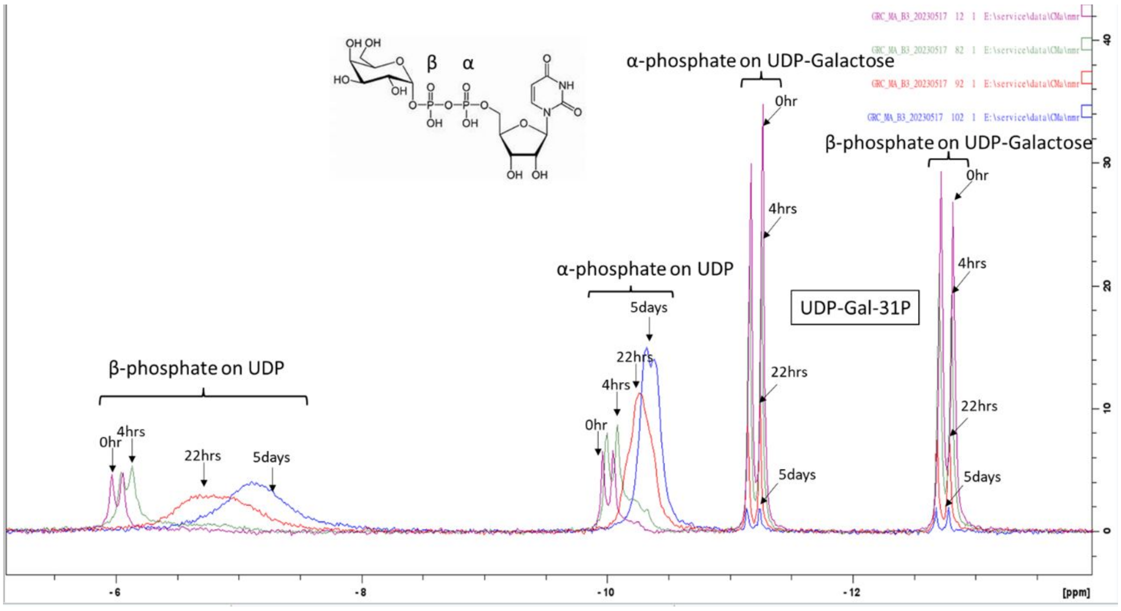

Figure S7. UDP-Galactose and UDP Phosphorus-31 signal by NMR spectroscopy. Phosphorus-31 nuclear magnetic resonance spectroscopy spectrum showing the range of P compounds is detected in the UDP-Galactose and UDP for this study. P-31 signals from the sample of β3GalT5 mixed with UDP-Galactose and Gb4 from time frame 0 hour to 5 days. The black arrows indicate different time points, the α-phosphate from UDP-Galactose, β-phosphate from UDP-Galactose, α-phosphate from UDP, and β-phosphate from UDP corresponding peaks were labeled. When UDP is released from β3GalT5 along with Mn2+, the peak becomes broader and right shifted.

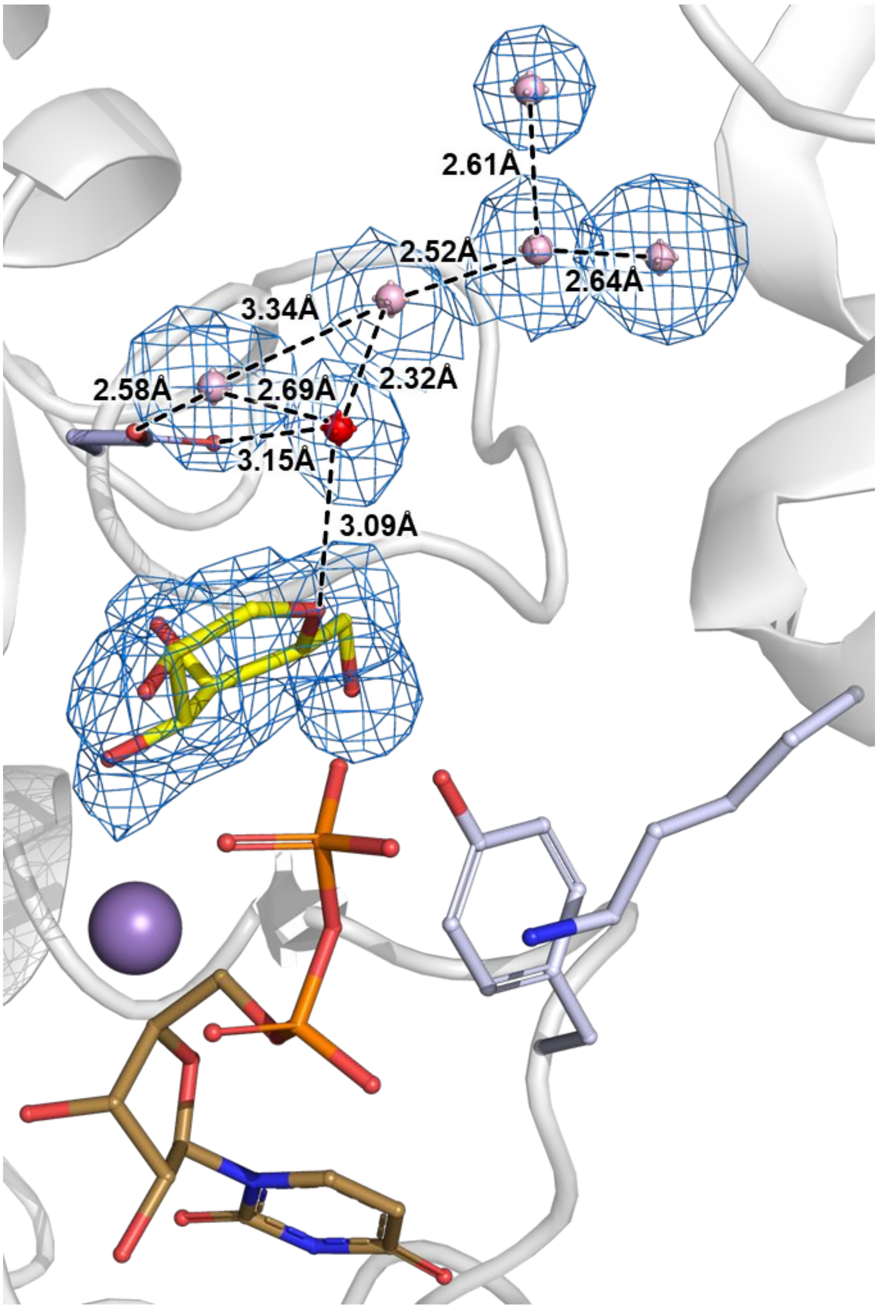

Figure S8. Electron density maps of the water network in β3GalT5:UDP:Gal (PDB: 8ZX9) structure. The structure is obtained by co-crystallizing β3GalT5 with UDP-Gal. The Polder omit maps were generated using PyMOL software, and F_o-F_c electron-density maps were contoured at 4σ. The key-interacting water molecule is colored red while other contributing water molecules are colored pink. Oxocarbenium ion-like galactose is colored yellow, and UDP is colored brown. The orientation of the figure was same as **Figure 4B**.

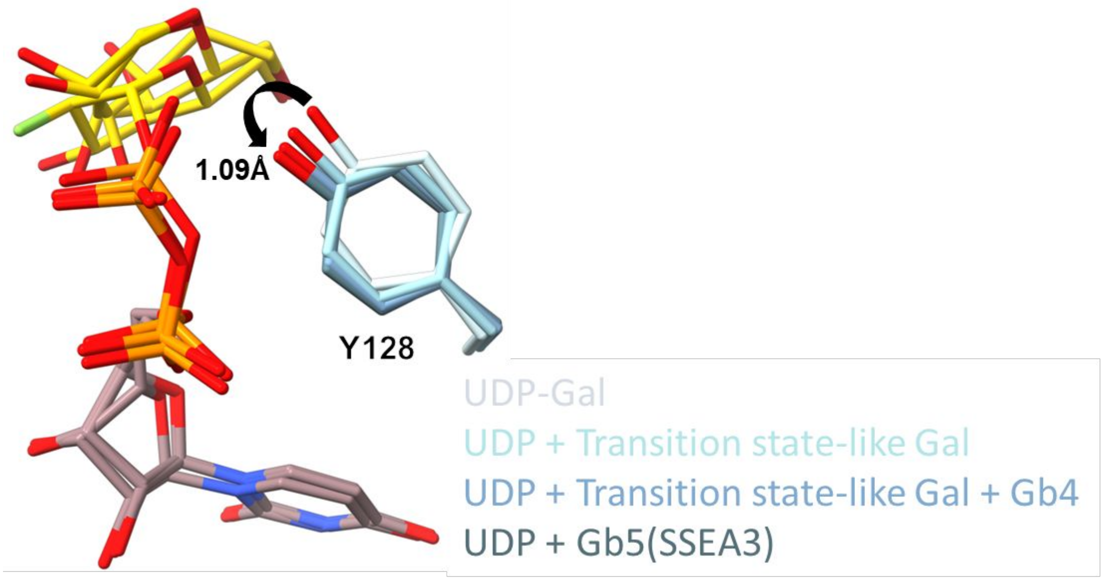

Figure S9. Tyrosine 128 conformational changes during reaction. The side chain of Tyr128 moves toward the β-phosphate of the leaving group UDP (in brown) during reaction with approximate distance of 1.09 Å from the UDP-2FGal bound structure (step 1) to Gb5 bound structure (step4).

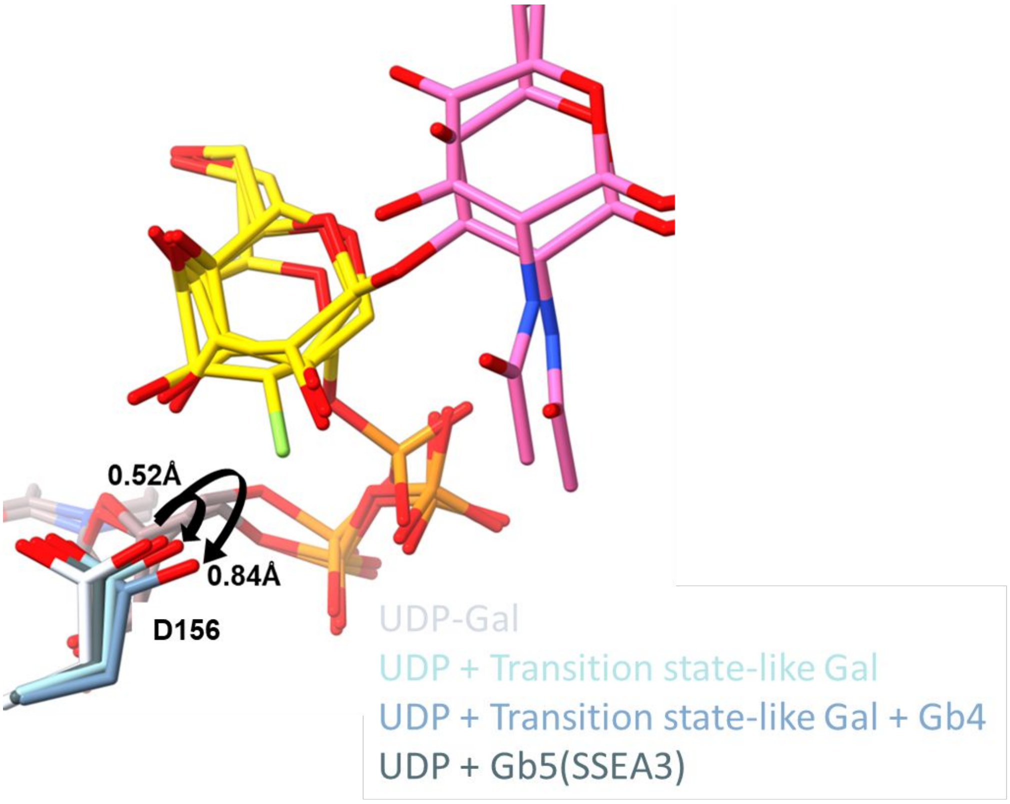

Figure S10. Aspartic acid 156 conformational changes during reaction. The side chain of Asp156 moves along with the donor galactose (in gold) during reaction with approximate movement of 0.52 Å from the UDP-2FGal bound structure (step 1) to galactose dissociated structure (step 2). Asp156 further moves approximately 0.32 Å when acceptor Gb4 (in pink) bound (step 3) making it 0.84 Å apart from step 1. Finally, when the product Gb5 forms (step 4), Asp156 returns to a position close to that observed in the UDP-2FGal bound structure.

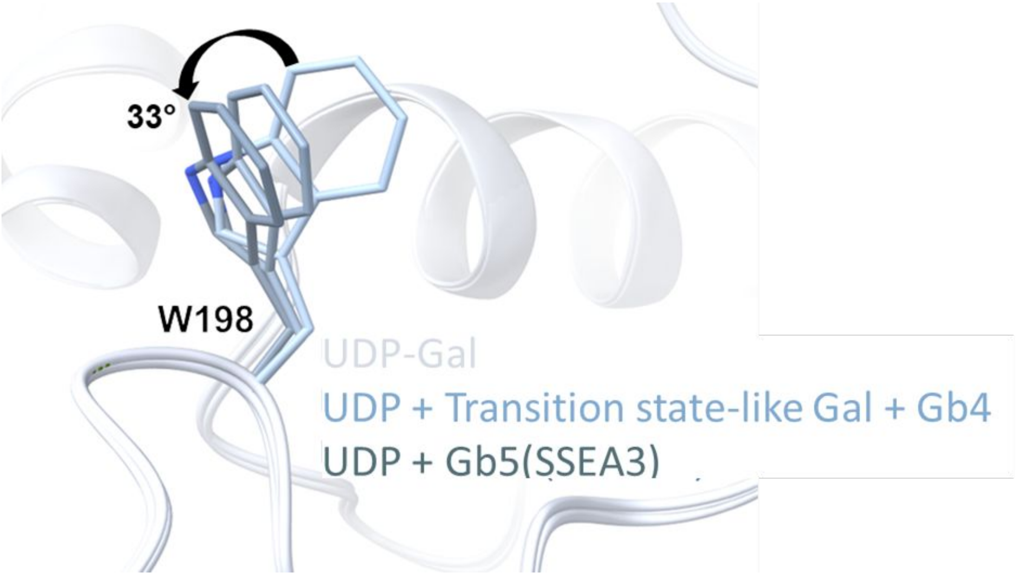

Figure S11. Tryptophan 198 conformational changes during reaction. The side chain of Trp198 movements throughout the reaction is like “lid on” and “lid off”. The angle difference between the initial UDP-2FGal bound structure (step 1) and the final product Gb5 bound structure (step 4) is approximately 33°.

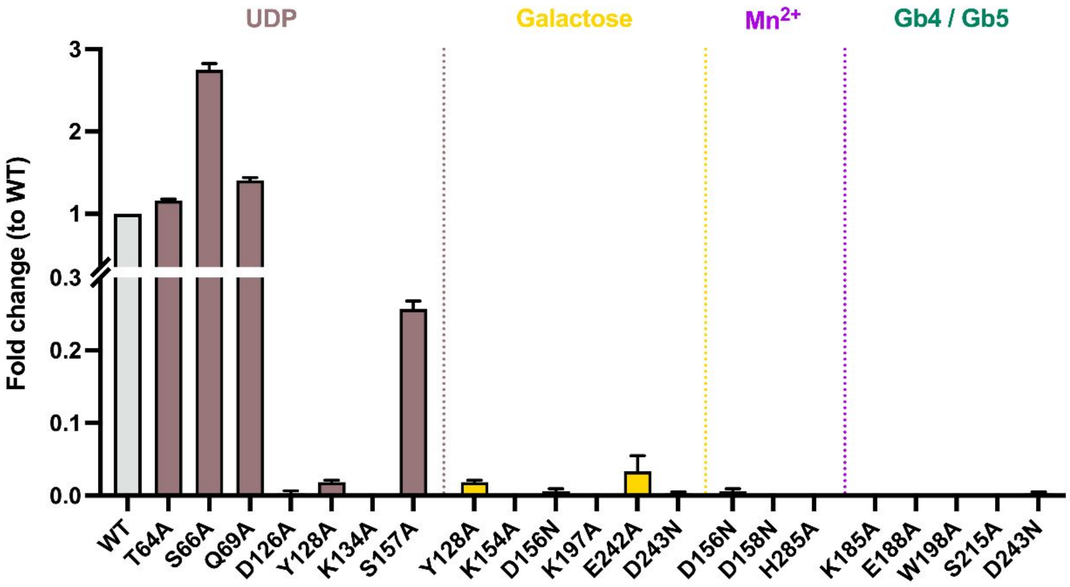

Figure S12. Assessment of β3GalT5 point mutants. The mutants are created to validate β3GalT5 key residue interactions, the activity of β3GalT5 is assessed using the UDP-Glo assay. The results are calculated relative to wild type (WT). The key residues are grouped according to their interactions as follows: UDP (in brown), donor galactose (in gold), divalent ion Mn^{2+} (in purple), and acceptor/final product (in pink). Some mutants appeared in two groups and they were: Y128A showed the interaction with both UDP and galactose moiety, D156N showed interaction with galactose or Mn2+ moiety, and D243N showed the interaction with the 3-OH and 4-OH of the terminal end GalNAc of Gb4-glycan and the β1,3-linkage between galactose and the Gb4-glycan.

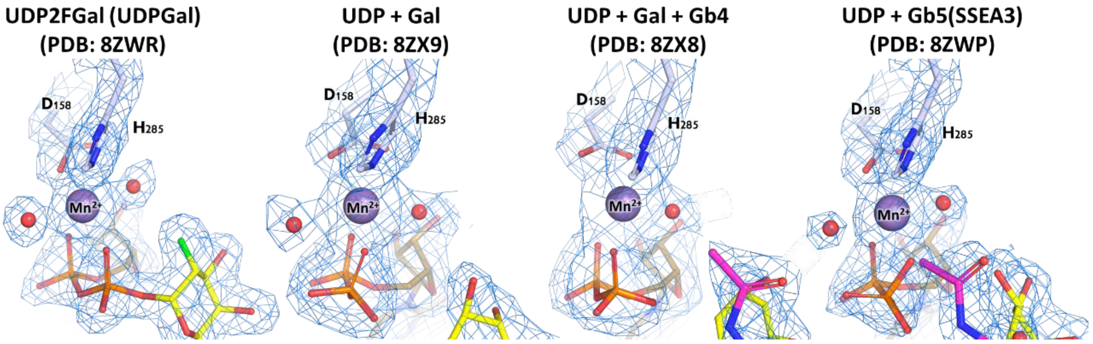

Figure S13. Electron density maps of the octahedral geometry of coordinated divalent ion Mn2+. The Mn^{2+} (colored purple) octahedral binding partners are Asp158 (from DXD motif), His285, diphosphate from UDP, and two or one water molecules (colored red). The $2F_o$-F_c density maps are contoured at 1.5σ for β3GalT5:UDP-2FGal structure (PDB: 8ZWR) and β3GalT5:UDP: Gb5/SSEA3 structure (PDB: 8ZWW), and are contoured at 1.0σ for β3GalT5:UDP:Gal structure (PDB: 8ZX9) and β3GalT5:UDP: Gal:Gb4 structure (PDB: 8ZX8).

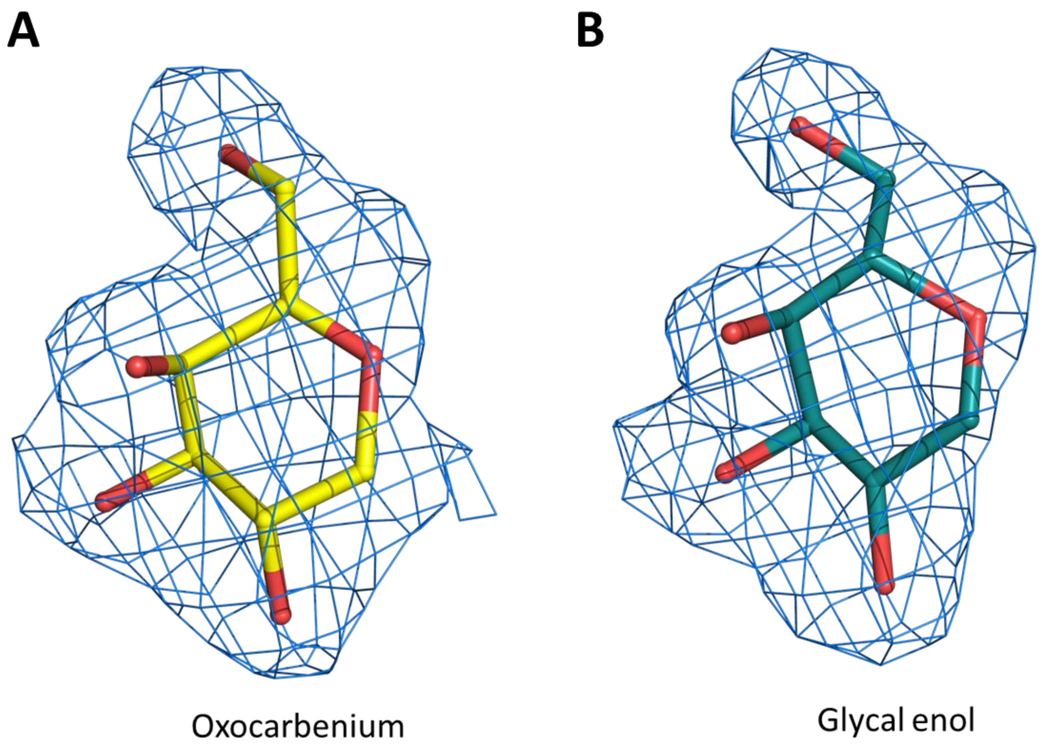

Figure S14. Electron density maps of the oxocarbenium-like galactose and the enol form of galactal. A. The Fo-Fc polder omit electron density map for oxocarbenium-like galactose from the β3GalT5:UDP:Gal complex structure (PDB: 8ZX9), contoured at 4σ and colored in yellow. **B.** The Fo-Fc polder omit electron density map for the enol form of galactal, refined using the PDB 8ZX9 dataset, contoured at 4σ and colored in deep teal.

Table S1. X-ray crystallographic data collection and structure refinement statistics (part 1)

	β3GalT5:UDP-2FGal	β3GalT5:UDP:Gal	β3GalT5:UDP:Gal:Gb4	β3GalT5:UDP:Gb4
**PDB code**	8ZWR	8ZX9	8ZX8	8ZWP
**Data collection**				
Space group	P 1 21 1	P 1 21 1	P 1 21 1	P 1 21 1
Cell dimensions				
a, *b*, *c* (Å)	47.74, 85.74, 87.73	47.98, 86.34, 87.51	47.80, 86.54, 87.64	47.65, 86.36, 87.19
α, β, γ (°)	90.00, 95.52, 90.00	90.00, 95.48, 90.00	90.00, 95.71, 90.00	90.00, 95.47, 90.00
Wavelength	1.00000	1.00000	1.00000	1.00000
Resolution (Å) [a]	50.00 - 1.94 (2.01 - 1.94) *	40.00 - 2.30 (2.38 - 2.30) *	50.00 - 2.40 (2.49 - 2.40) *	50.00 - 1.86 (1.93 - 1.86) *
R_{sym} or R_{merge} [a, b]	0.082 (0.676)	0.080 (0.551)	0.130 (0.742)	0.067 (0.724)
$I / \sigma I$ [a]	14.93 (1.18)	19.12 (2.14)	9.85 (1.12)	21.95 (2.13)
Completeness (%)[a]	94.9 (74.3)	98.3 (89.0)	98.0 (85.0)	99.4 (97.7)
Redundancy [a]	4.1 (3.1)	4.4 (3.5)	3.8 (2.6)	5.7 (5.4)
**Refinement**				
Resolution (Å) [a]	24.97 - 2.05 (2.12 - 2.05) *	26.63 - 2.30 (2.38 - 2.30) *	30.72 - 2.40 (2.49 - 2.40) *	28.84 - 1.86 (1.93 - 1.86) *
No. reflections [a]	40705 (2665)	28685 (1682)	23999 (1042)	58604 (5703)
R_{work} / R_{free} [c, d]	0.1825/0.2168	0.1793/0.2125	0.1798/0.2270	0.1950/0.2175
No. atoms				
Protein	4417	4394	4394	4412
Ligand/ion	301	301	386	362
Water	393	198	126	249
B-factors				
Protein	34.49	42.72	49.41	43.56
Ligand/ion	51.07	65.39	88.05	67.92
Water	34.48	39.22	40.84	41.29
R.m.s. deviations				
Bond lengths (Å)	0.004	0.007	0.005	0.015
Bond angles (°)	1.05	1.02	0.80	1.43

Table S1. X-ray crystallographic data collection and structure refinement statistics (part 2)

	β3GalT5:UDP:Gb 5/SSEA3	β3GalT5:UDP-Gal:GlcNac-β1,3-Gal-OMe	β3GalT5:UDP-Gal:GlcNAc-β1,3-GalNAc-α-Thr	β3GalT5:UDP-Gal:Man-β1,6-Man
**PDB code**	8ZWW	8ZWY	8ZX3	8ZX2
**Data collection**				
Space group	P 1 21 1	P 1 21 1	P 1 21 1	P 1 21 1
Cell dimensions				
a, *b*, *c* (Å)	47.71, 86.13, 87.61	47.65, 86.70, 87.00	47.54, 86.37, 86.89	47.60, 86.50, 86.22
α, β, γ (°)	90.00, 95.68, 90.00	90.00, 95.25, 90.00	90.00, 95.26, 90.00	90.00, 94.86, 90.00
Wavelength	1.00000	1.00000	1.00000	1.00000
Resolution (Å) [a]	50.00 - 1.87 (1.94 - 1.87) *	50.00 - 1.95 (2.02 - 1.95)	50.00 - 1.87 (1.94 - 1.87) *	50.00 – 1.86 (1.93 – 1.86) *
R_{sym} or R_{merge} [a,b]	0.122 (1.706)	0.086 (0.362)	0.046 (0.542)	0.067 (0.724)
$I / \sigma I$ [a]	10.19 (0.43)	16.20 (3.45)	28.02 (1.86)	21.76 (2.15)
Completeness (%)[a]	95.6 (71.8)	98.1 (89.0)	94.1 (73.9)	99.4 (97.7)
Redundancy [a]	3.9 (2.5)	4.9 (4.3)	4.0 (3.2)	5.7 (5.4)
**Refinement**				
Resolution (Å) [a]	28.86 - 2.20 (2.28 - 2.20) *	43.32 - 1.95 (2.02 - 1.95) *	25.70 - 2.10 (2.18 - 2.10) *	27.19 - 2.40 (2.49 - 2.40) *
No. reflections [a]	34270 (2599)	47592 (3613)	39030 (3113)	24938 (1763)
R_{work} / R_{free} [c, d]	0.1827/0.2295	0.1887/0.2275	0.1969/0.2286	0.1885/0.2349
No. atoms				
Protein	4422	4396	4470	4390
Ligand/ion	350	323	290	311
Water	285	261	269	145
B-factors				
Protein	37.45	30.95	39.32	41.97
Ligand/ion	61.82	43.50	74.25	75.09
Water	35.04	28.13	34.71	34.90
R.m.s. deviations				
Bond lengths (Å)	0.004	0.017	0.014	0.005
Bond angles (°)	0.71	1.38	1.61	0.83

*Highest resolution shell is shown in parenthesis.

[a]Values in parentheses are for the highest resolution shell.

[b]$R_{merge} = \Sigma|I_i - \langle I_i \rangle|\Sigma I_i$, I_i is the average intensity value of the equivalent reflections.

[c]$R_{work} = \Sigma hkl||F_{obs}|-|F_{calc}||/\Sigma hkl|F_{obs}|$.

[d]R_{free} was calculated from 5% of data randomly excluded data from refinement.

Reference

(1) Otwinowski Z Fau - Minor, W.; Minor, W. Processing of X-ray diffraction data collected in oscillation mode. (1557-7988 (Electronic)).

(2) Terwilliger, T. C.; Adams, P. D.; Read, R. J.; McCoy, A. J.; Moriarty, N. W.; Grosse-Kunstleve, R. W.; Afonine, P. V.; Zwart, P. H.; Hung, L. W. Decision-making in structure solution using Bayesian estimates of map quality: the PHENIX AutoSol wizard. *Acta Crystallogr D Biol Crystallogr* **2009**, *65* (Pt 6), 582-601.

(3) McCoy Aj Fau - Grosse-Kunstleve, R. W.; Grosse-Kunstleve Rw Fau - Adams, P. D.; Adams Pd Fau - Winn, M. D.; Winn Md Fau - Storoni, L. C.; Storoni Lc Fau - Read, R. J.; Read, R. J. Phaser crystallographic software. (0021-8898 (Print)).

(4) Emsley, P.; Cowtan, K. Coot: model-building tools for molecular graphics. *Acta Crystallogr D Biol Crystallogr* **2004**, *60* (Pt 12 Pt 1), 2126-2132.

(5) Afonine, P. V.; Grosse-Kunstleve, R. W.; Echols, N.; Headd, J. J.; Moriarty, N. W.; Mustyakimov, M.; Terwilliger, T. C.; Urzhumtsev, A.; Zwart, P. H.; Adams, P. D. Towards automated crystallographic structure refinement with phenix.refine. *Acta Crystallogr D Biol Crystallogr* **2012**, *68* (Pt 4), 352-367.

(6) Vagin, A. A.; Steiner, R. A.; Lebedev, A. A.; Potterton, L.; McNicholas, S.; Long, F.; Murshudov, G. N. REFMAC5 dictionary: organization of prior chemical knowledge and guidelines for its use. *Acta Crystallogr D Biol Crystallogr* **2004**, *60* (Pt 12 Pt 1), 2184-2195.

(7) DeLano, W. L. Use of PYMOL as a communications tool for molecular science. *Abstr Pap Am Chem S* **2004**, *228*, U313-U314.

(8) Meng, E. C.; Goddard, T. D.; Pettersen, E. F.; Couch, G. S.; Pearson, Z. J.; Morris, J. H.; Ferrin, T. E. UCSF ChimeraX: Tools for structure building and analysis. *Protein Sci* **2023**, *32* (11), e4792.
